# Time-Resolved Raman Spectroscopy of Nitrate Electroreduction Reveals Dynamic Interfacial Adsorbate Evolution

**DOI:** 10.1021/jacsau.6c01050

**Published:** 2026-10-10

**Authors:** Gabriel F. Costa, Jaxiry S. Barroso-Martínez, Dídac-Armand Fenoll, Conor Brennan-Pollak, Max García-Melchor, María Escudero-Escribano

**Affiliations:** † Catalan Institute of Nanoscience and Nanotechnology (ICN2), CSIC and Barcelona Institute of Science and Technology, UAB Campus, 08193 Bellaterra, Barcelona, Spain; ‡ 138819 CIC energiGUNE, Parque Tecnológico, C/Albert Einstein 48, 01510 Vitoria-Gasteiz, Spain; § School of Chemistry, Trinity College Dublin, Dublin 2, Ireland; ∥ IKERBASQUE, Basque Foundation for Science, Plaza de Euskadi 5, 48009 Bilbao, Spain; ⊥ Catalan Institution for Research and Advanced Studies (ICREA), Pg. Lluís Companys 23, 08010 Barcelona, Spain

**Keywords:** electrocatalysis, nitrate reduction, time-resolved spectroscopy, surface-enhanced Raman spectroscopy, adsorption dynamics, electrified interfaces, density functional theory

## Abstract

Nitrate electroreduction offers a pathway for coupling wastewater remediation with sustainable ammonia production, yet its mechanistic details under operating conditions remain poorly understood. In this work, we directly resolve the dynamic evolution of nitrate-derived intermediates at electrified interfaces, addressing a key limitation in understanding dynamic reaction pathways. We employ time-resolved surface-enhanced Raman spectroscopy (TR-SERS) to monitor the interfacial behavior of NO_3_^–^ and NO_2_^–^ during nitrate reduction on roughened silver electrodes and observe potential-dependent vibrational shifts attributed to systematic changes in adsorption modes under increasingly cathodic potentials. Density functional theory calculations further support the vibrational assignments and provide insight into preferred binding motifs across the applied potential range. By tracking the time evolution of adsorbates, this approach provides insights into the sequence of adsorption and transformation steps on silver. The combined experimental and computational analysis contributes to a molecular-level understanding of nitrate electroreduction and provides a framework for probing dynamic reaction intermediates in nitrogen conversion electrocatalysis.

## Graphical abstract

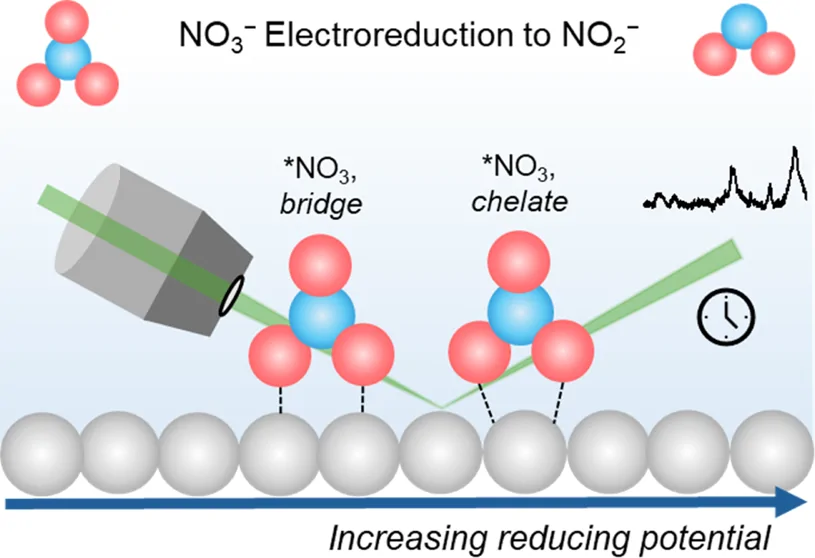

## 1 Introduction

Nitrate (NO_3_^–^) accumulation in aquatic systems caused by agricultural runoff and industrial wastewater poses serious ecological and human health risks.^1–7^ These concerns have intensified efforts to develop effective remediation strategies. Electrocatalytic nitrate reduction (NO_3_RR) has emerged as a promising approach, offering an alternative to conventional treatments such as ion exchange and adsorption.^1,4^ When powered by renewable electricity and using water as the proton source, NO_3_RR can convert NO_3_^–^ into benign N_2_ or value-added NH_3_.^6,8^ Recent advances in catalyst design,^9^ electrolyte,^7,10–12^ and device engineering^13^ have enabled high yields and stable operation at elevated current densities, underscoring the potential of NO_3_RR for sustainable nitrogen management.

Despite this progress, NO_3_RR remains mechanistically complex. The reaction proceeds through multiple electron-transfer and proton-coupled electron transfer (PCET) steps, involves several surface-bound intermediates, and competes strongly with the hydrogen evolution reaction (HER).^4,14–17^ Although numerous mechanistic proposals have been advanced, many key intermediates have been inferred indirectly. In particular, the initial reduction of nitrate to nitrite is widely proposed to constitute a kinetically significant and, in many cases, rate-limiting step in NO_3_RR.^18^ Identifying the structure, stability, and reactivity of species such as nitrate and nitrite is therefore essential for understanding this early transformation and its influence on downstream reactivity.^16,19,20^ Such understanding is also relevant to related processes, including C–N coupling reactions^21^ and electrochemical urea synthesis from the coelectrolysis of CO_2_ and NO_3_^–^.^16,22,23^ Time-resolved spectroscopic evidence of adsorbed species under reaction conditions is thus necessary to validate and refine proposed mechanisms.^19,20,24^

Among transition metals, silver provides a particularly suitable platform for interrogating nitrate-derived adsorbates involved in this early reduction step.^25^ Silver exhibits appreciable activity for NO_3_RR, selectively producing nitrite (NO_2_^–^) at relatively low overpotentials while maintaining comparatively low HER activity. Moreover, because silver does not efficiently catalyze ammonia formation under mild conditions,^25^ nitrate-derived intermediates can persist longer on the surface than on more active metals such as Cu and Rh,^21,26,27^ facilitating spectroscopic detection. In addition to its catalytic properties, silver is an established substrate for surface-enhanced Raman spectroscopy (SERS).^28,29^ In particular, nanostructured silver exhibits strong plasmonic enhancement, enabling sensitive detection of surface-bound species.^30^ Consequently, Ag-based electrodes provide a combined catalytic and spectroscopic platform for probing potential-dependent adsorption of nitrate and nitrite under electrochemical conditions.^31,32^

Previous Raman studies have reported nitrogen-containing intermediates during nitrate reduction; however, these measurements have largely been performed under steady-state conditions.^33–35^ While such approaches provide valuable structural information, they offer limited insight into the dynamic evolution of adsorbates as electrode potential and surface coverage change. As a result, transient variations in adsorption geometry, competitive hydrogen adsorption, and coverage-dependent interactions may significantly influence reaction pathways yet remain obscure in steady-state measurements. While time-resolved Raman spectroscopy has been used to monitor potential-dependent interfacial transformations during CO_2_ electroreduction,^19,20^ comparable investigations of nitrate electroreduction are still lacking, particularly for the nitrate-to-nitrite conversion step.

To address this limitation, we employ time-resolved surface-enhanced Raman spectroscopy (TR-SERS) to monitor nitrate and nitrite adsorption under dynamic, operando electrochemical conditions on a roughened Ag electrode. Combined with cyclic voltammetry, TR-SERS resolves potential-dependent shifts of NO_3_^–^ vibrational modes that reflect changes in adsorption geometry with increasingly cathodic polarization. Here, “time-resolved” refers to the continuous acquisition of Raman spectra during cyclic voltammetry rather than under steady-state potentiostatic conditions, enabling the dynamic evolution of interfacial adsorbates to be monitored as the electrode potential continuously changes.^19,20^ Spectra were acquired with a temporal resolution of 4 s while scanning at 5 mV s^–1^, corresponding to a potential change of approximately 20 mV per acquisition. The observed spectral evolution indicates that nitrate-binding configurations are highly sensitive to both applied potential and surface coverage. To further rationalize these observations, we performed density functional theory (DFT) calculations for adsorbed NO_3_^–^ (*NO_3_, where * denotes an active surface site) and NO_2_^–^ (*NO_2_) on a Ag(111) surface model. Ag(111) was selected as the primary model because it is the most compact and thermodynamically stable silver facet and therefore provides a suitable representation of terrace sites on the flat Ag electrode. To account for the structural heterogeneity introduced by the roughening procedure, two additional defective surface models containing undercoordinated Ag sites were also considered. Together, these models provide a useful approximation to the range of adsorption environments expected across both the flat and roughened Ag electrodes while acknowledging that further reconstruction may occur under *operando* conditions. The predicted resting states show that increasing hydrogen coverage modulates nitrate binding through hydrogen-bond interactions and weakens its adsorption.^36^ Comparison of computed vibrational frequencies for multiple adsorption geometries with experimental Raman features enables the assignment of potential-dependent binding motifs and provides insight into how the local environment influences the nitrate-to-nitrite conversion step.

The combination of TR-SERS and DFT modeling provides time-resolved, molecular-level insights into dynamic interfacial phenomena during the electroreduction of nitrate to nitrite on silver. The focus is placed on the nitrate-to-nitrite reduction step, which is frequently implicated in governing the overall reaction kinetics. Likewise, emphasis is placed on understanding how the interfacial environment and adsorption configuration evolve with applied potential rather than on evaluating overall nitrate-removal performance or the formation of downstream reduction products. Collectively, these findings establish a framework for correlating potential-dependent adsorption geometries with reaction progression and illustrate how interface-sensitive spectroscopy, supported by atomistic modeling, can clarify key elementary steps in electrocatalytic nitrate reduction.

## 2 Results and Discussion

### 2.1 Electrochemical Characterization of Silver for Nitrate and Nitrite Reduction

We first examined the electrochemical response of nitrate and nitrite reduction on both a flat and an oxidation-roughened silver disc, the latter serving as the substrate for Raman measurements, to assess its suitability for TR-SERS. The preparation of the roughened Ag electrode is described in the Experimental Methods section in the Supporting Information, while Figure S1 shows the roughening protocol and the corresponding voltammetric response. We present the electrochemically active surface area (ECSA), determined by Pb underpotential deposition (upd), in Figure S2, and optical and scanning electron micrographs acquired before and after roughening are shown in Figures S3 and S4, respectively. These characterizations confirm the substantial modification of the Ag surface induced by the oxidation–reduction treatment. Consistent with this behavior, previous studies of SERS-active Ag substrates prepared by similar roughening procedures have reported nanometric porous morphologies that enhance local electromagnetic fields and promote Raman signal amplification.^37,38^

Figure 1 shows the cyclic voltammograms (CVs) for NO_3_^–^ and NO_2_^–^ reduction on the flat (Figure 1a) and roughened (Figure 1b) silver electrodes. At cathodic potentials more negative than −0.10 V vs RHE, we observe two reduction features for NO_3_^–^ (I–II), whereas a single dominant feature appears for NO_2_^–^ reduction (III). These features are consistent with literature assignments, in which the cathodic peaks in NO_3_RR are attributed to the reduction of NO_3_^–^ to NO_2_^–^ (eq 1) using different proton donors (H_2_O and H_2_PO_4_^–^).^25,39,40^ This assignment is further supported by the high Faradaic efficiency toward NO_2_^–^ observed over the potential range from −0.2 to −0.5 V vs RHE (Figures S5 and S6), indicating that NO_2_^–^ is the predominant nitrogen-containing product under these conditions. An additional reduction wave emerges beyond −0.60 V vs RHE, which we attribute to the onset of hydrogen evolution, consistent with the relatively late HER activity of Ag (gray traces in Figure 1).

1 $${NO}_{3}^{-}+{2e}^{-}+H_{2}O\to {NO}_{2}^{-}+{2OH}^{-}$$

**Figure 1:**
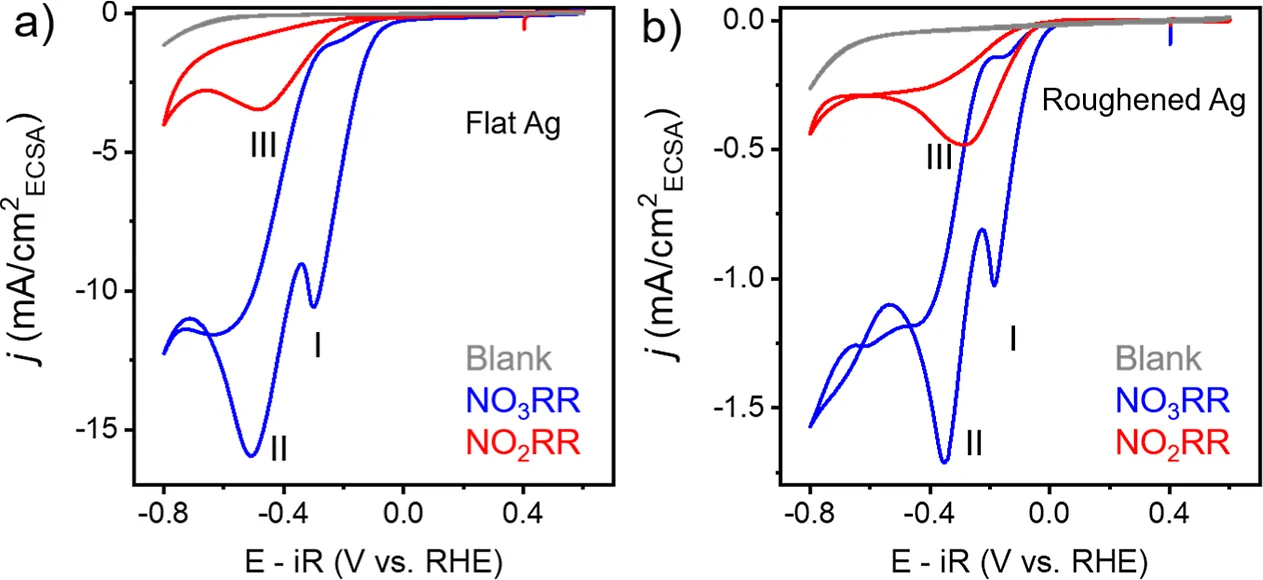
Cyclic voltammetric characterization of silver electrodes for nitrate to nitrite reduction. Cyclic voltammograms (CVs) of blank, NO_3_RR, and NO_2_RR on (a) flat and (b) oxidation-roughened silver discs. A reversible hydrogen electrode (RHE) and a Ag wire were used as the reference and counter electrodes, respectively. CVs were recorded at 20 mV s^–1^ in 0.1 M K_*x*_H_3–*x*_PO_4_ buffer (pH 7) (gray traces) containing either 0.1 M KNO_3_ (blue traces) or 0.1 M KNO_2_ (red traces).

From Figure 1b, the onset potentials for NO_3_RR and NO_2_RR in 0.1 mol L^–1^ phosphate buffer (pH 7) are approximately 0.00 V and −0.10 V vs RHE, respectively, consistent with previous reports.^41^ To facilitate mechanistic investigation of adsorbed nitrate species, experiments were performed at a sufficiently high nitrate concentration (0.1 mol L^–1^) to maintain measurable surface populations throughout the potential range examined. Compared to the flat polycrystalline Ag electrode (Figure 1a), the roughened Ag electrode exhibits slightly less cathodic onset potentials for both NO_3_RR and NO_2_RR (Figure 1b). This trend is consistent with prior observations for oxide-derived Ag electrocatalysts, where oxidation–reduction cycling generates undercoordinated sites and grain boundaries that modify adsorption energetics.^41–44^ Surface restructuring under operating conditions may therefore contribute to the observed shifts, as discussed in Section 2.5.

Notably, the roughened Ag electrode displays an order of magnitude lower current density than that of the flat surface. We note that this current density is ECSA-normalized, determined from the charge associated with Pb underpotential deposition (Pb upd), as described below, and therefore provides an estimate of the surface area that is electrochemically accessible to Pb deposition.^45,46^ This behavior likely reflects a combination of mass transport limitations and local electric field effects within the nanostructured morphology, which can restrict effective diffusion and create heterogeneous interfacial environments.^37,38,47^ Figure S4 shows scanning electron microscopy (SEM) images of the flat (Figure S4a,b) and roughened (Figure S4c,d) Ag electrodes, revealing the formation of cavities following the ORC treatment. These recessed features contribute to the increased ECSA determined by Pb upd but may not be equally accessible to nitrate under the NO_3_RR conditions. Consequently, mass-transport limitations within these cavities may hinder nitrate replenishment and result in a lower effective surface area for nitrate reduction than that estimated from Pb upd.^48,49^ Nevertheless, the roughened electrode provides sufficient electrochemical activity within the potential window relevant to the nitrate-to-nitrite conversion step, with comparable Faradaic efficiencies toward nitrite (Figure S6) making it suitable for TR-SERS measurements.

### 2.2 In Situ Time-Resolved Surface Enhanced Raman Spectra of Nitrate Electrochemical Reduction on Silver

We performed in situ TR-SERS measurements during three consecutive CV cycles of NO_3_RR on the roughened Ag electrode, with the potential scanned from +0.60 to −0.80 V vs RHE. Figure 2a correlates the applied potential (black trace) with the corresponding current density for the third cycle (blue trace), which is representative of the spectroscopic and electrochemical behavior observed throughout the measurements. We show the potential-dependent Raman response recorded during this cycle as a heatmap in Figure 2b, where the dotted white lines indicate the seven representative spectra (I–VII) shown in Figure 2c. Control measurements obtained in a blank electrolyte and during NO_2_^–^ reduction are provided in Figures S7 and S8, respectively.

**Figure 2:**
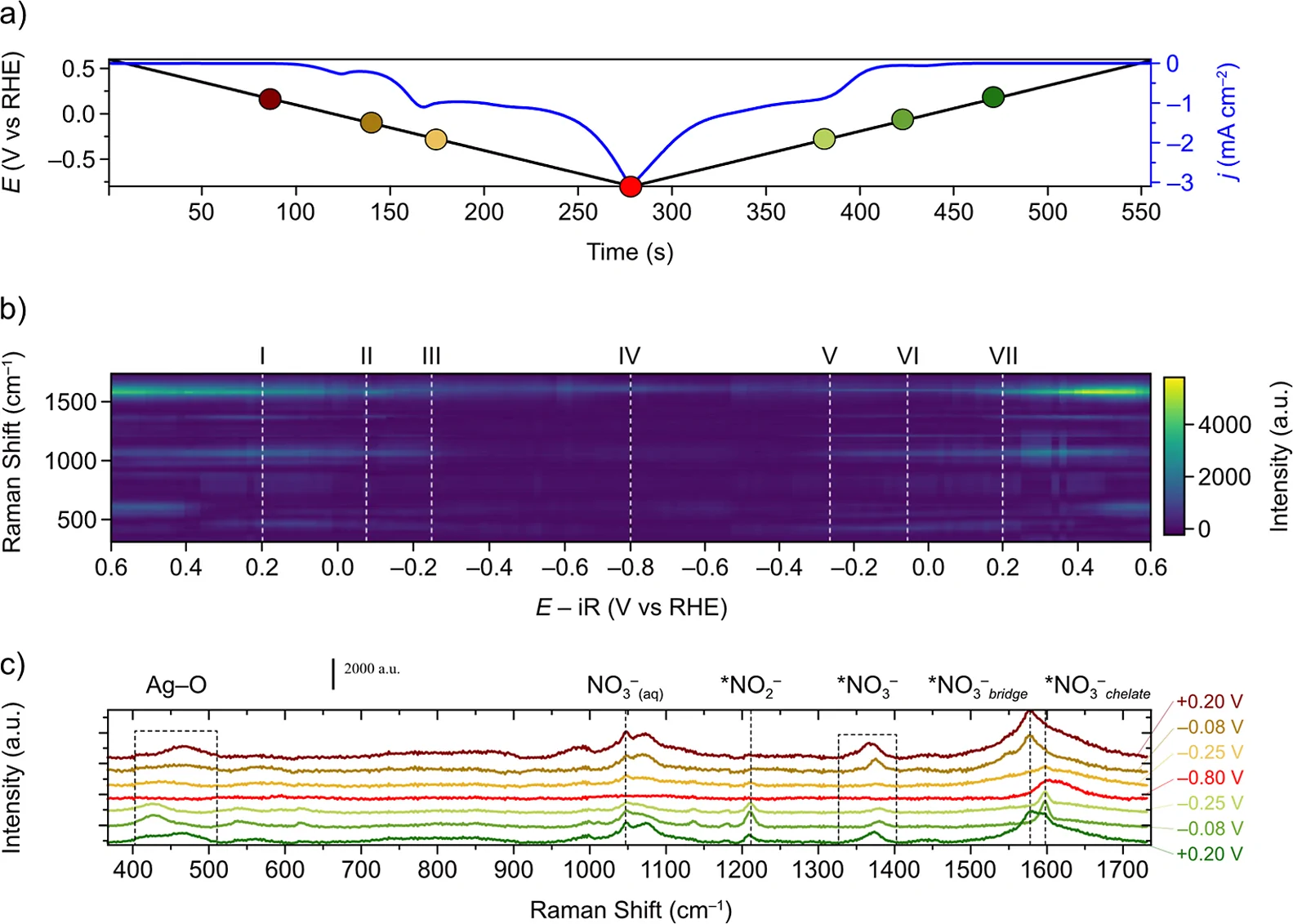
In situ time-resolved surface-enhanced Raman spectroscopy during nitrate electroreduction on roughened silver. (a) Applied potential (black trace) and corresponding current density (blue trace) during the third cyclic voltammetry (CV) cycle of NO_3_RR. (b) Potential-dependent Raman heatmap recorded during the same cycle. Dotted white lines labeled I–VII indicate the spectra displayed in the panel below. (c) Representative Raman spectra acquired at +0.20 V, −0.08 V, −0.25 V, and −0.80 V vs RHE during the cathodic scan (I–IV) and at −0.25 V, −0.08 V, and +0.20 V vs RHE during the anodic scan (V–VII). Peaks of interest are marked with dashed traces. CVs were recorded at 5 mV s^–1^ from +0.60 to −0.80 V vs RHE using roughened Ag as the working electrode, a reversible hydrogen electrode as the reference electrode, and a Ag wire as the counter electrode. The electrolyte consisted of 0.1 M K_*x*_H_3–*x*_PO_4_ (pH 7) containing 0.1 M KNO_3_. Raman spectra were collected using a 532 nm laser (50 mW) with a 4 s acquisition time and a 50× objective lens.

As the potential is swept cathodically, new vibrational features emerge that we attribute primarily to adsorbed *NO_3_ and *NO_2_ species associated with the initial nitrate-to-nitrite conversion step. In the low-frequency region, we observe a band below 500 cm^–1^ that shifts systematically with applied potential, red-shifting (i.e., shifting to lower frequencies) during the cathodic scan and blue-shifting (i.e., shifting to higher frequencies) during the anodic sweep. This feature is consistent with Ag–O stretching modes of O-coordinated anions at the interface, arising from *NO_3_ and/or coordinated phosphate species from the supporting electrolyte, both of which give rise to a comparable band in this region, as confirmed by control measurements performed in the blank electrolyte (Figure S7). The potential-dependent shift reflects the response of these Ag–O interactions to the changing interfacial field.^33^ This behavior is consistent with computed low-energy vibrational modes of adsorbed *NO_3_ in which *O–N–O bending couples to vertical displacement of the coordinating Ag atom (*vide infra*). Together, these trends suggest progressive weakening of Ag–O interactions under increasingly cathodic bias, potentially preceding partial *NO_3_ or phosphate desorption.

We assign the band at 1047 cm^–1^ to the symmetric stretching mode $\left({\mathrm{\nu ̅}}_{1}\right)$ of aqueous NO_3_^–^, whereas the feature between 1373 and 1380 cm^–1^ is assigned to the antisymmetric stretching mode $\left({\mathrm{\nu ̅}}_{3}\right)$ of adsorbed *NO_3_.^33^ Given the observed potential-sensitive dynamics, we attribute the peaks centered at 1580 and 1596 cm^–1^ to differently coordinated adsorbed *NO_3_ species and assign to bidentate bridge (*NO_3,bridge_) and chelate (*NO_3,chelate_) configurations, respectively.^50^

Under more strongly cathodic conditions (≤−0.40 V vs RHE), the spectra become dominated by a broad band centered at 1610 cm^–1^, assigned to interfacial water (Figure 2c, yellow trace).^51^ The attenuation of well-defined nitrate-related features in this potential region indicates that the interfacial vibrational response becomes governed by water and hydrogen adsorption, as *NO_3_ and *NO_2_ are progressively desorbed under increasingly cathodic conditions.

In addition to nitrate-related modes, Figure 2c reveals the potential-dependent evolution of a *NO_2_ band centered at 1209 cm^–1^.^34^ We observe a weaker *NO_2_ signal near −0.40 V vs RHE during the cathodic sweep, corresponding to the region immediately following the second reduction feature in the CV (Figure 1b, peak II), whereas the band becomes more prominent during the anodic sweep (Figure 2c, green traces). This trend is consistent with *NO_2_ being generated during the cathodic scan but remaining predominantly in solution as NO_2_^–^ under strongly reducing conditions. Upon reversing the potential, changes in interfacial adsorption equilibria promote NO_2_^–^ adsorption, resulting in an enhanced Raman response at 1209 cm^–1^ corresponding to *NO_2_. We show the evolution of the intensity of this band with applied potential in Figure S9b.

### 2.3 Dynamics of Nitrate Adsorption and Desorption

To probe nitrate adsorption dynamics more quantitatively, we monitored the evolution of the band centered between 1360 and 1380 cm^–1^ during NO_3_RR (Figure 3). As a reference, comparison with Raman spectra of solid and aqueous potassium nitrate (Figure S10) confirms that the 1047 cm^–1^ peak corresponds to the symmetric stretching mode of NO_3_^–^ and not *NO_3_. In the solid salt (Figure S10, black trace), splitting of the antisymmetric stretching mode $\left({\mathrm{\nu ̅}}_{3}\right)$ into peaks at 1342 and 1359 cm^–1^ arises from symmetry reduction within the crystal lattice, from *D*_2*h*_ in the free ion to approximately *C*_2*v*_,^32^ due to a slight distortion that renders one N–O bond inequivalent relative to the other two. By analogy, a comparable symmetry lowering is expected upon nitrate adsorption at the silver interface, giving rise to distinct features in the 1360–1380 cm^–1^ region, as we discuss below.

**Figure 3:**
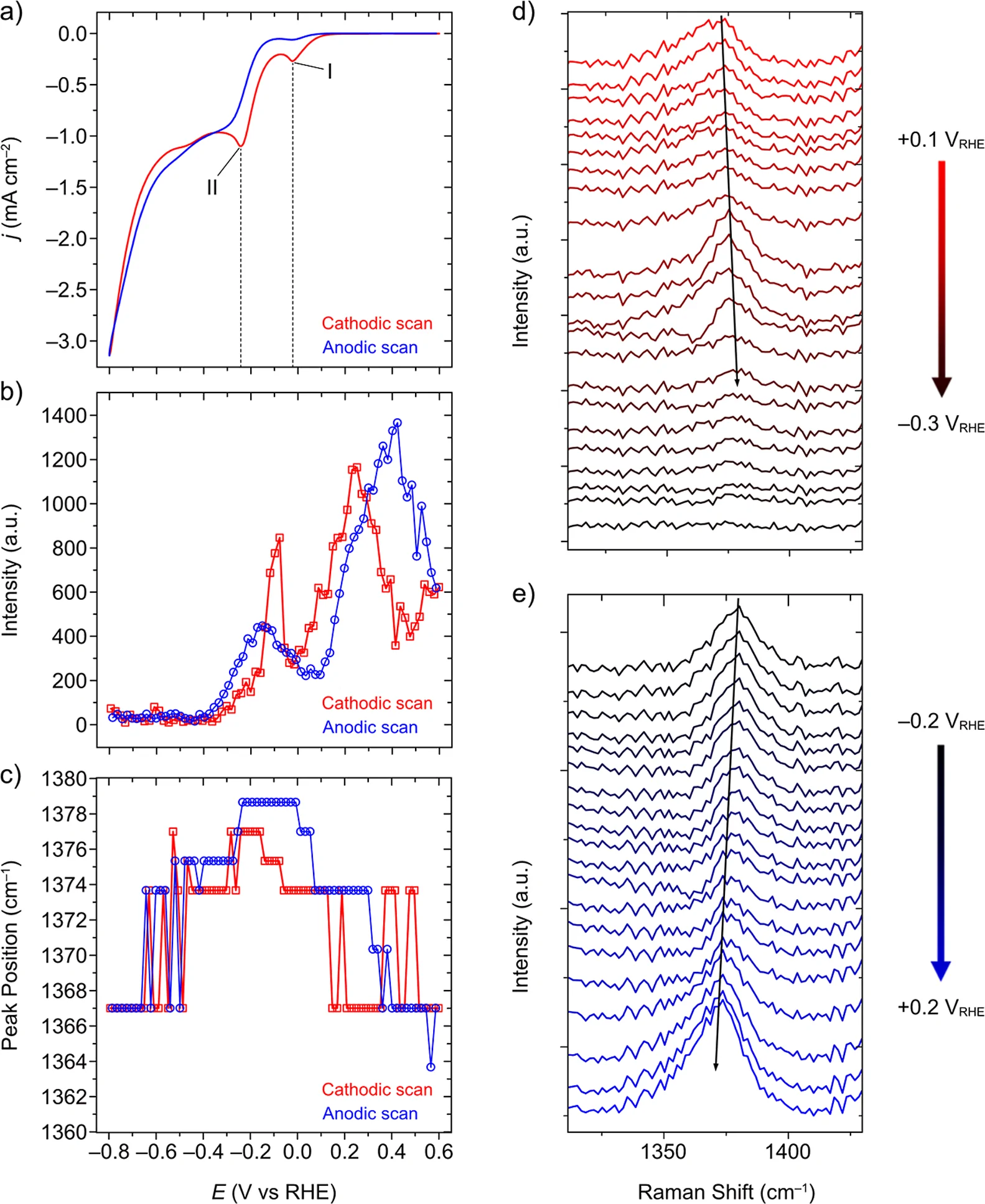
Dynamics of nitrate adsorption during NO_3_RR on roughened silver. (a) Cyclic voltammogram (third cycle) recorded during TR-SERS measurements. (b) Potential-dependent evolution of the intensity of the band centered between 1360 and 1380 cm^–1^. (c) Corresponding peak position as a function of applied potential. Red and blue traces denote cathodic and anodic scans, respectively. (d) Representative Raman spectra acquired during the cathodic scan between +0.10 and −0.30 V vs RHE. (e) Representative Raman spectra acquired during the anodic scan between −0.20 and +0.20 V vs RHE. CVs were recorded at 5 mV s^–1^ using roughened Ag as the working electrode, a reversible hydrogen electrode as the reference electrode, and a Ag wire as the counter electrode in 0.1 M K_*x*_H_3–*x*_PO_4_ (pH 7) containing 0.1 M KNO_3_. Raman spectra were collected using a 532 nm laser (50 mW) with a 4 s acquisition time and a 50× objective lens.

We show the third CV cycle during which Raman spectra were collected in Figure 3a. The two cathodic reduction features coincide closely with maxima in the intensity of the 1360–1380 cm^–1^ band (Figure 3b), indicating that changes in nitrate adsorption are strongly coupled to the electrochemical reduction events. During the anodic sweep, the intensity of this band increases further at more positive potentials, reaching higher values than those observed during the cathodic scan. This asymmetry is due to the accumulation of nitrite formed by nitrate reduction during the cathodic sweep, followed by partial readsorption of nitrate and interfacial reorganization upon reversal of the potential.

Beyond intensity changes, the position of the 1360–1380 cm^–1^ band also varies systematically with applied potential (Figure 3c), exhibiting maxima at +0.25 and −0.07 V vs RHE during the cathodic scan (red trace, Figure 3b) and at −0.13 and +0.42 V vs RHE during the anodic sweep (blue trace, Figure 3b). During the cathodic scan, this band undergoes a pronounced blue shift between +0.10 and −0.30 V vs RHE (Figure 3c, red trace; Figure 3d, black arrow). Such behavior is consistent with changes in metal–adsorbate interaction strength and local interfacial charge density.^35,52^ Specifically, the progressive red shift of the 450 cm^–1^ band indicates a weakening of the Ag–O interaction at increasingly reducing potentials. This weakening reduces back-donation to the adsorbate nitrate species, strengthening the N–O bond associated with the noncoordinated oxygen atom and giving rise to the observed blue shift. During the anodic scan, the opposite trend is observed, with the 1360–1380 cm^–1^ band exhibiting a red shift between −0.2 and +0.2 V vs RHE (Figure 3e), consistent with a strengthening of the Ag–O interaction. In addition, hydrogen bonding between interfacial hydrogen and coadsorbed *NO_3_ may further influence the vibrational frequency by modifying the effective reduced mass and local coordination environment.

### 2.4 Different Modes of Nitrate Adsorption

To further elucidate the coexistence of different nitrate adsorption geometries, we analyzed the peaks at 1580 and 1596 cm^–1^. We assign these features to *NO_3,bridge_ and *NO_3,chelate_ configurations, respectively.^37,38,50^ The 1500–1600 cm^–1^ region contains multiple N–O and interfacial water stretching modes that can shift depending on coordination and the local interfacial environment.^50,53–55^ Consequently, reliable assignment requires consideration of potential-dependent behavior and interfacial conditions rather than frequency values alone.

In the present case, the *NO_3,bridge_ and *NO_3,chelate_ assignments are supported by complementary observations: (i) their coexistence with the spectroscopic feature discussed in the previous subsection (i.e., the blue-shift from 1360 to 1380 cm^–1^), (ii) systematic and reversible potential-dependent shifts, (iii) distinct intensity evolution of the two bands, and (iv) previous assignments for those adsorbates in heterogeneous catalysis.^53,54^ In addition, the observed trends are consistent with adsorption geometries calculated by DFT under relevant electrochemical conditions, as discussed in Section 2.5.

We illustrate the evolution of these bands during electrochemical cycling in Figure 4. In the +0.10 to −0.30 V vs RHE window of the cathodic scan (Figure 3a), distinct reduction features coincide with pronounced spectral changes in the 1520–1610 cm^–1^ region. We summarize the corresponding evolution of band intensities and peak positions in Figure 4a,b, revealing that, over much of this potential range, the *NO_3,bridge_ band at 1580 cm^–1^ exhibits higher intensity than both the *NO_3,chelate_ band at 1596 cm^–1^ and the interfacial water band at 1610 cm^–1^.

**Figure 4:**
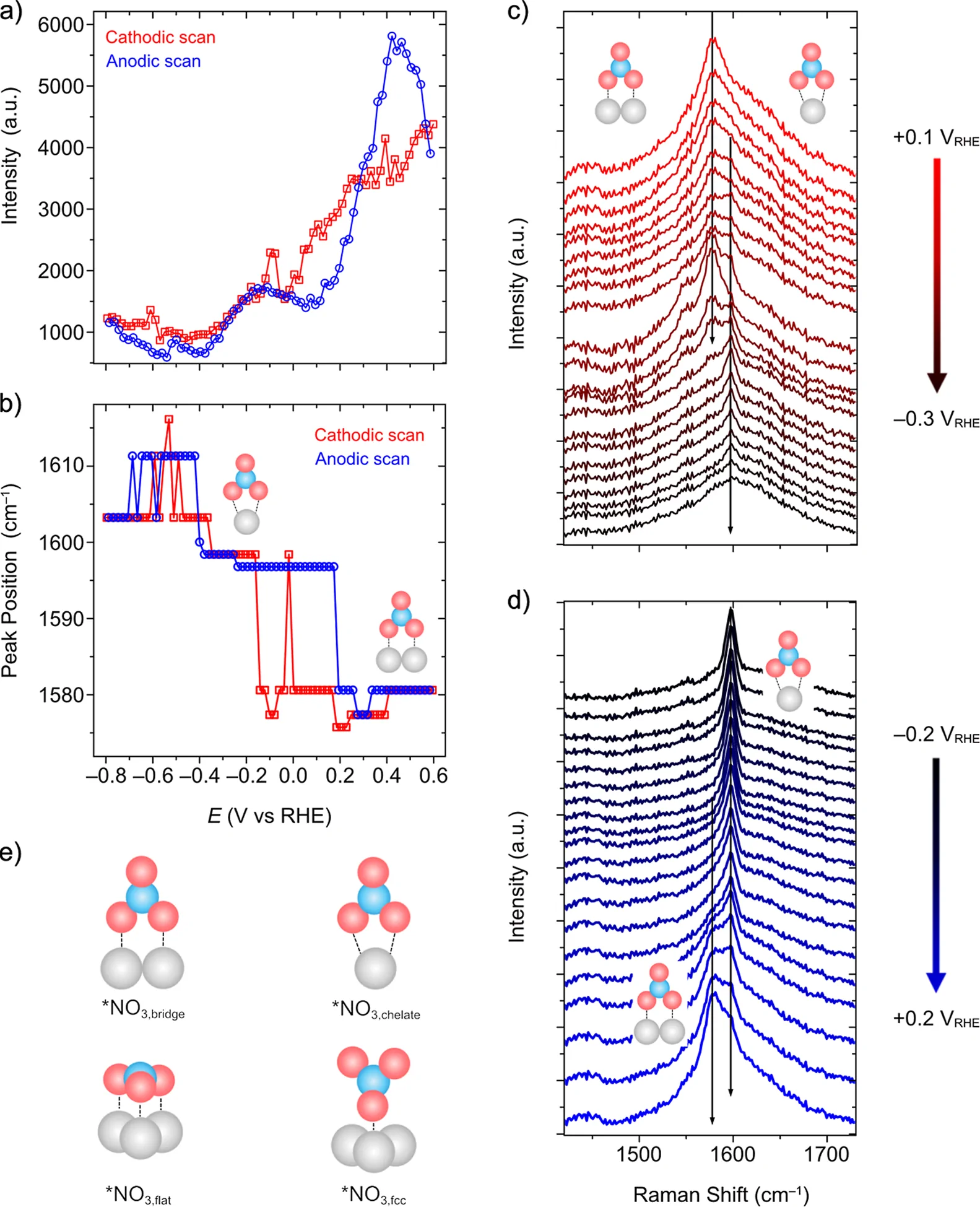
Modes of nitrate adsorption during NO_3_RR on roughened silver. (a) Potential-dependent evolution of Raman band intensities in the 1520–1610 cm^–1^ region. (b) Corresponding peak positions as a function of applied potential. Red and blue traces denote cathodic and anodic scans, respectively. (c) Representative Raman spectra acquired during the cathodic scan between +0.10 and −0.30 V vs RHE. (d) Representative Raman spectra acquired during the anodic scan between −0.20 and +0.20 V vs RHE. Insets illustrate the proposed bridge (*NO_3,bridge_) and chelate (*NO_3,chelate_) nitrate adsorption geometries associated with the 1580 and 1596 cm^–1^ bands, respectively. (e) DFT-optimized geometries of representative nitrate-binding configurations on the flat Ag(111) surface, including bridge (*NO_3,bridge_), chelate (*NO_3,chelate_), tridentate flat (*NO_3,flat_), and monodentate fcc (*NO_3,fcc_). Raman spectra were collected using a 532 nm laser (50 mW) with a 4 s acquisition time and a 50× lens.

As the potential becomes increasingly cathodic within this window (Figure 4b,c, red trace), the peak position shifts progressively from values characteristic of the *NO_3,bridge_ configuration toward those associated with *NO_3,chelate_ coordination at 1596 cm^–1^. This systematic shift indicates a redistribution of nitrate adsorption geometry under increasingly reducing conditions. During the anodic sweep, the spectral evolution reverses, and the band position shifts back toward values characteristic of the *NO_3,bridge_ configuration in the −0.20 to +0.20 V vs RHE range (Figure 4a–d, blue trace), consistent with partial reversibility of the adsorption geometry. We show in Figure 4e a scheme of some representative binding modes of nitrate.

Importantly, the emergence of the NO_2_^–^ band at approximately −0.25 V vs RHE in Figure 2c occurs within the same potential window in which the *NO_3,chelate_ configuration becomes increasingly prominent. This temporal and potential correlation suggests that the transition from *NO_3,bridge_ to *NO_3,chelate_ coordination precedes, and may facilitate, the nitrate-to-nitrite reduction. Although the present data do not directly resolve the bond-breaking step, they indicate that changes in nitrate-binding geometry are closely coupled to the onset of nitrite formation.

### 2.5 DFT Analysis of Nitrate and Nitrite Adsorption on Flat and Defective Ag Surfaces

To provide atomistic insights into the observed spectral trends and support the corresponding assignments, we investigated the adsorption of *NO_3_ and *NO_2_ on flat and defective Ag surfaces using periodic DFT calculations. Details of the computational methodology are provided in Section S2 of the Supporting Information.

In addition to the flat Ag(111) surface model, we constructed two defective models to probe the influence of local undercoordination on adsorption and vibrational properties: Ag_1_, consisting of a single Ag adatom on an fcc site, and Ag_3_, a three-atom aggregate occupying contiguous fcc sites (Figure 5a). These motifs reduce the number of available terrace fcc sites while introducing undercoordinated binding sites, thereby increasing the diversity of adsorption configurations accessible to the *H and *NO_*x*_ species. The Ag_1_ and Ag_3_ models were therefore used primarily to assess how localized deviations from an ideal Ag(111) terrace affect the adsorption geometries and vibrational frequencies of *NO_3_ and *NO_2_. To complement these localized defect models with a more extended representation of the step sites that may be present on roughened Ag, we additionally considered a stepped Ag(211) surface, evaluating *NO_3_ and *NO_2_ adsorption on both bare and H-covered surfaces (*vide infra*). Together, Ag(111), Ag_1_/Ag_3_, and Ag(211) provide complementary models for assessing the influence of terrace sites, localized defects, and step sites, respectively, on the adsorption and vibrational properties of nitrate- and nitrite-derived species.

**Figure 5:**
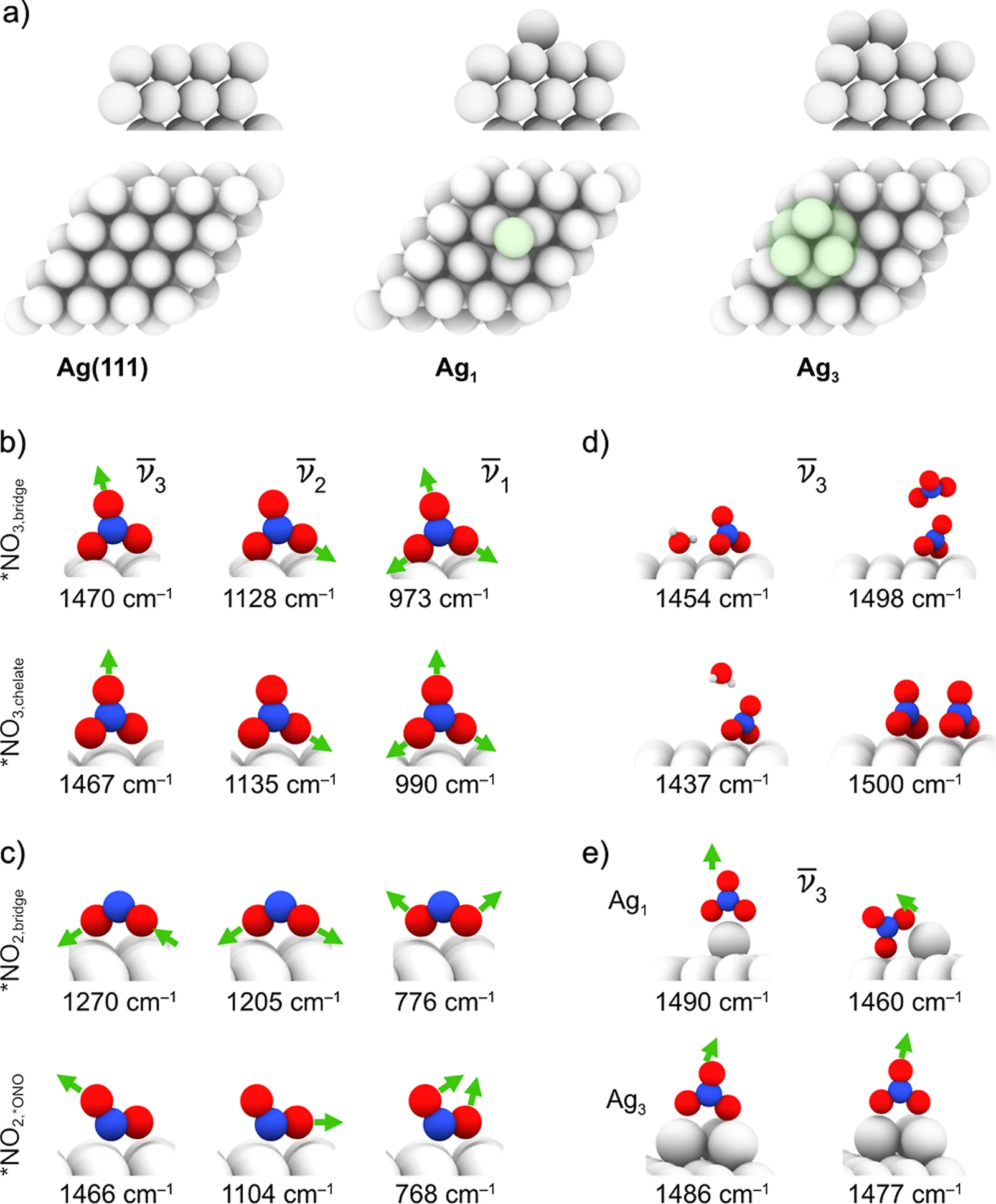
DFT analysis of nitrate and nitrite adsorption on Ag(111) and localized defect models. (a) Side and top views of optimized flat Ag(111), Ag_1_, and Ag_3_ surface models used to assess the influence of the local surface structure (defect sites are highlighted in green). (b) Calculated vibrational wavenumbers (in cm^–1^) and displacement vectors (green arrows) for the three highest-frequency ( ${\mathrm{\nu ̅}}_{3},{\mathrm{\nu ̅}}_{2}$ , and ${\mathrm{\nu ̅}}_{1}$ ) normal modes of *NO_3_ in bidentate bridge (*NO_3,bridge_) and chelate (*NO_3,chelate_) configurations on the flat Ag(111) surface. (c) Corresponding normal modes for *NO_2_ in bridge (*NO_2,bridge_) and *ONO (*NO_2,*ONO_) configurations on the flat Ag(111) surface. (d) Influence of coadsorption on the ${\mathrm{\nu ̅}}_{3}$ stretching mode of *NO_3,bridge_ on the flat Ag(111) surface. (e) Calculated ${\mathrm{\nu ̅}}_{3}$ stretching frequencies of *NO_3_ at undercoordinated sites of the defective Ag_1_ and Ag_3_ surface models.

While these surface models capture key aspects of the structural diversity of the Ag electrode, the surface state under electrochemical conditions is also governed by adsorbate coverage.^56^ Increasing evidence indicates that electrocatalytic interfaces should be considered dynamic with the preferred surface composition and structure evolving with the reaction conditions. Species originating from the electrolyte can also alter surface coverages, block available sites, and modify catalytic pathways.^36,57^ In particular, hydrogen coverage can play a critical role in electrochemical hydrogenation reactions under neutral and alkaline conditions.^57^

To establish the potential-dependent surface state of Ag electrodes and its impact on nitrate and nitrite adsorption, the hydrogen surface coverages on Ag(111) and Ag_3_ were explicitly evaluated (Figure S12). On flat Ag(111), hydrogen binds weakly even at fcc sites (Figure S12a), requiring potentials more negative than −0.60 V vs RHE to reach 0.25 ML (monolayer) *H coverage. On defective Ag_3_ (Figure S12b), formation of 0.25 ML *H is further delayed by approximately 0.10 V relative to flat Ag(111). This trend is consistent with the experimentally observed delayed onset of the HER on roughened Ag (Figure 1) and illustrates how local surface structure can modify the potential-dependent stability of adsorbed hydrogen.

Within the conventional constant-charge (CC) DFT framework combined with the computational hydrogen electrode (CHE),^58^ increasing hydrogen coverage systematically weakens the adsorption of both *NO_3_ and *NO_2_ (see Computational Methods in the Supporting Information for further details). At 0 V vs RHE, both species bind only weakly to bare Ag(111), with ${\Delta G}_{{*}_{{NO}_{3,bridge}}}=-0.03\;eV$ and ${\Delta G}_{{*}_{{NO}_{2,bridge}}}=-0.01\;eV$ , while at 0.25 ML *H, adsorption becomes unfavorable ( ${\Delta G}_{{*}_{0.25H,{*}_{{NO}_{3,bridge}}}}=+0.55\;eV$ and ${\Delta G}_{{*}_{0.25H,{*}_{{NO}_{2,bridge}}}}=+0.80\;eV$ Figure S13b). These results indicate a strong coupling between increasing *H coverage and the stability of nitrate- and nitrite-derived surface species.

However, because the CC/CHE treatment does not explicitly account for potential-dependent changes in surface charge, the quantitative stability ranges of these coadsorbed states warrant further assessment. To assess these potential-dependent surface-charging effects, we additionally performed constant-potential (CP) calculations for selected *H-, *NO_3_-, and *NO_2_-containing Ag(111) surface states using a grand-canonical charge-interpolation scheme.^56,59^ The CC/CHE and CP treatments yield closely comparable adsorption energetics for 0.25 ML *H (Figure S14), consistent with our previous systematic analysis showing a close agreement between the two approaches for *H adsorption on Ag(111) over a broader range of H coverages.^56^ We therefore retain the CC/CHE description of the potential-dependent evolution of *H coverage while using CP calculations to assess surface-charging effects on the nitrate- and nitrite-containing states. In contrast to *H, *NO_3_ and *NO_2_ exhibit appreciable non-Nernstian contributions under CP conditions, resulting in quantitative changes in their potential-dependent stability relative to the CC/CHE predictions. In particular, CP calculations predict *NO_3_ coadsorption with 0.25 ML *H to remain thermodynamically favorable down to approximately −0.60 V vs RHE. At more cathodic potentials, the CC/CHE surface-coverage analysis (Figure S12a) predicts the stabilization of denser *H coverages, which eventually disfavors *NO_3_ coadsorption and favors nitrate desorption. The use of CC/CHE for these higher *H coverages is supported by the close CC/CP agreement obtained here at 0.25 ML *H and by our previous benchmark showing a similarly close agreement for *H adsorption on Ag(111) up to 1 ML.^56^ Conversely, CP treatment does not modify the predicted stability range of *NO_2_ significantly compared to CC/CHE: NO_2_ remains favorably adsorbed only until approximately −0.01 V vs RHE. However, its coadsorption with *H does become favorable at ca. −0.65 V vs RHE, unlike the result from the CC/CHE prediction, which suggested potentials below −1.00 V vs RHE were needed to achieve stable *H, *NO_2_ coverages (certainly well above the cathodic potential where HER would dominate). Thus, *NO_3_ and *NO_2_ both become destabilized as denser *H coverages become thermodynamically favored, with non-Faradaic effects favoring the adsorption of *NO_3_ over the CC/CHE description. Overall, the CP results presented herein retain the overall trend toward H-dominated surface states under strongly cathodic conditions.

Hydrogen coverage also alters the relative stabilities of nitrate adsorption geometries. As fcc sites become progressively occupied by *H, the *NO_3,bridge_ and *NO_3,chelate_ configurations (Figure 5) approach similar stability at 0.25 ML *H, with adsorption Gibbs energies of +0.55 and +0.52 eV, respectively. This near-degeneracy is consistent with the potential-dependent redistribution between the Raman features assigned to *NO_3,bridge_ and *NO_3,chelate_ in the TR-SERS measurements described in Section 2.4.

To further assess the spectroscopic assignments, we computed the vibrational normal modes for several *NO_3_ adsorption geometries. Because the calculated frequencies are sensitive to both the adsorption geometry and the local coordination environment, we use the DFT results primarily to evaluate mode character and relative frequency trends rather than to reproduce small experimental shifts quantitatively. The three highest-frequency modes for *NO_3,bridge_ and *NO_3,chelate_ are shown in Figure 5b, together with the corresponding modes for *NO_2,bridge_ and *NO_2,*ONO_ configurations (Figure 5c). In addition to the most stable adsorption structures, we evaluated alternative *NO_3_-binding modes on bare Ag(111) and defective surfaces to rationalize the larger frequency shifts observed experimentally and assess the range of vibrational responses associated with different local adsorption environments.

At 0 V vs RHE on the bare surface, *NO_3_ stability decreases in the order bridge ( ${\Delta G}_{{*}_{{NO}_{3,bridge}}}=-0.03\;eV$ , Figure 5b) > tridentate flat ( ${\Delta G}_{{*}_{{NO}_{3,flat}}}=0.01\;eV$ , Figure S15a) > monodentate fcc ( ${\Delta G}_{{*}_{{NO}_{3,fcc}}}=+0.13\;eV$ , Figure S15b) > chelate ( ${\Delta G}_{{*}_{{NO}_{3,chelate}}}=+0.77\;eV$ , Figure 5b). The computed ${\mathrm{\nu ̅}}_{3}$ asymmetric stretching frequency spans a broad range across these geometries, with variations approaching 300 cm^–1^ (Figure S16a,b). The highest-frequency mode corresponds to the asymmetric stretching of the two outward-oriented N–O bonds in the *NO_3,fcc_ configuration, with a computed frequency of 1548 cm^–1^, in good agreement with the experimental value of 1565 cm^–1^.^53,54^

Lower-energy modes span a narrower frequency range but exhibit distinct displacement patterns depending on their coordination to the surface. In contrast to ${\mathrm{\nu ̅}}_{3}$ , the symmetric stretching mode ${\mathrm{\nu ̅}}_{1}$ follows an opposite trend across adsorption geometries (Figure 5b). These differences are reflected in the computed normal modes of *NO_3,bridge_ and *NO_3,chelate_, with the bridge configuration exhibiting a higher-frequency ${\mathrm{\nu ̅}}_{3}$ mode but lower-frequency ${\mathrm{\nu ̅}}_{2}$ and ${\mathrm{\nu ̅}}_{1}$ modes (Figure 5b). The computed frequency separation between *NO_3,bridge_ and *NO_3,chelate_ $\left({\Delta \mathrm{\nu ̅}}_{DFT}=3\;{cm}^{-1}\right)$ is smaller than the experimentally observed separation (ca. 10 cm^–1^) but is consistent with its direction and the relative ordering of the two features on bare Ag(111). Together with the distinct mode characteristics of the two adsorption geometries, this trend is consistent with the assignment of the experimentally observed features to *NO_3,bridge_ and *NO_3,chelate_.

To further rationalize the differences between computed and experimental frequencies, we evaluated the influence of coadsorption and the local interfacial environment (Figure 5d). The highest-frequency modes involve N–O stretches oriented away from the surface and are particularly sensitive to neighboring species, which can modify the local bonding environment and thereby perturb the corresponding vibrational frequencies. To probe the influence of neighboring water, we considered two distinct H_2_O coadsorption configurations, with H_2_O positioned either near the outward-oriented O atom of NO_3_, allowing direct hydrogen-bonding interactions, or closer to the Ag–O–N interfacial region. These configurations were selected to sample distinct local interactions with adsorbate nitrate rather than to provide an exhaustive description of the interfacial water structure. Consistent with their different local environments, the two H_2_O configurations induce red shifts of approximately 30 and 16 cm^–1^, respectively, in the relevant nitrate vibrational mode, whereas interactions with neighboring *NO_3_ adsorbates or uncoordinated nitrate anions produce blue shifts of comparable magnitude (Figure 5d). Importantly, the calculated ${\mathrm{\nu ̅}}_{3}$ separation between the bridge and chelate configurations increases from 3 cm^–1^ on bare Ag(111) to 17 cm^–1^ in the presence of neighboring *H_2_O, whereas the *NO_3_–*NO_3_ coadsorption model yields a small inversion of −2 cm^–1^. These variations demonstrate that both the magnitude and, for sufficiently small splittings, the ordering of the calculated frequencies are sensitive to the immediate coadsorption environment. Consequently, differences of only a few cm^–1^ should not be interpreted quantitatively across different interfacial configurations. More generally, these competing local interactions can contribute to the broad distribution of ${\mathrm{\nu ̅}}_{3}$ frequencies and help rationalize the spectral broadening observed experimentally under reaction conditions (Figure 2).

Surface morphology introduces an additional source of variability by modifying the local coordination environment of adsorbed species. In particular, adsorption at undercoordinated defect sites such as Ag_1_ and Ag_3_ (Figure 5e) weakens *NO_3_ binding relative to flat Ag(111), resulting in blue shifts of the corresponding vibrational modes. A shift of approximately 20 cm^–1^ is predicted for the *NO_3,chelate_ configuration on Ag_1_, with a smaller shift on Ag_3_, consistent with differences in local coordination (3-fold vs 5-fold). These effects further illustrate the sensitivity of the calculated frequencies to surface structure and help rationalize differences between spectra obtained on roughened electrodes and those computed for idealized surface models.

Finally, we performed an analogous analysis for *NO_2_ adsorption on the Ag_1_ (Figure S18a) and Ag_3_ (Figure S18b) defective models. Agreement between computed and experimental frequencies is generally better than for nitrate, particularly for the symmetric stretching mode near 1200 cm^–1^. Nonetheless, the computed frequencies remain sensitive to adsorption geometry and the presence of low-coordination surface sites. Simulated spectra based on off-resonance Raman activities are provided in Figure S18, with additional discussion in Section S2.4.

Despite the substantially different surface morphology and binding-site geometries, the main trends in *NO_3_ and *NO_2_ adsorption identified on Ag(111) are largely retained on a stepped Ag(211) surface. At 0 V vs RHE on the bare surface, adsorption of both nitrogen-containing species is thermodynamically favorable. *NO_3_ binds only weakly $\left({\Delta G}_{{*}_{{NO}_{3}}}=-0.03\;eV\right)$ in a geometry distinct from the bridge and chelate configurations identified on Ag(111), whereas *NO_2_ adsorption at a bridge site is substantially more favorable $\left({\Delta G}_{{*}_{{NO}_{2,bridge}}}=-0.37\;eV\right)$ . We additionally evaluated *NO_3_ and *NO_2_ adsorption on Ag(211) in the presence of 0.25 ML coadsorbed *H, representative of the H-covered states favored under reducing conditions. At this coverage, the adsorption of both nitrogen-containing species is thermodynamically unfavorable at 0 V vs RHE, with adsorption Gibbs energies of ${\Delta G}_{{*}_{{NO}_{3}}}=+0.22\;eV$ and ${\Delta G}_{{*}_{{NO}_{2}}}=+1.00\;eV$ . The calculated vibrational frequencies on Ag(211) are also comparable to those obtained on Ag(111) (Table S1). The largest differences are found for the ${\mathrm{\nu ̅}}_{3}$ and ${\mathrm{\nu ̅}}_{1}$ modes of *NO_3_, which are blue- and red-shifted by 16 cm^–1^, respectively, on Ag(211) relative to Ag(111), consistent with the different nitrate adsorption geometries on the two surfaces. Smaller differences are obtained for *NO_2_, which adopts similar bridge configurations on both facets: the ${\mathrm{\nu ̅}}_{3}$ frequency is unchanged, while the ${\mathrm{\nu ̅}}_{2}$ and ${\mathrm{\nu ̅}}_{1}$ modes are blue-shifted by only 4 and 3 cm^–1^, respectively. Thus, inclusion of the stepped Ag(211) model does not provide closer quantitative agreement with the experimental frequencies than Ag(111), although the overall vibrational characteristics remain comparable across the terrace and stepped surface models.

Overall, vibrations involving N–O bonds oriented away from the surface exhibit greater sensitivity to the local interfacial environment, whereas modes involving surface-coordinated N–O bonds are comparatively less sensitive and generally show closer agreement with the experiment, based on the smaller changes these modes experienced when considering the coadsorption simulations. The calculated mode characteristics and relative frequency trends are consistent with the experimentally observed Raman features, while the coadsorption and surface models demonstrate that the precise frequencies can vary substantially with the local environment. Together, these results support the proposed spectroscopic assignments while emphasizing that small calculated frequency differences should be interpreted qualitatively rather than as quantitative predictions of the experimental peak separations.

The sensitivity of the adsorption energetics and vibrational frequencies to the local surface structure also raises the possibility that potential-dependent changes in Ag morphology could influence the observed interfacial behavior. Because the present surface models represent selected local coordination environments rather than the dynamic evolution of the electrode, they do not explicitly capture such a restructuring. Potential-dependent changes in the relative abundance of terrace, step, and other undercoordinated sites could therefore further modify the relative stability and vibrational signatures of nitrate adsorption configurations, providing an additional source of interfacial heterogeneity beyond adsorbate coverage and coadsorption. Although such effects cannot be excluded by the present calculations, the reproducibility of the TR-SERS response over three consecutive CV cycles, including the peak positions, relative intensities, and potential-dependent trends, suggests that any morphological evolution occurring over the experimental time scale does not produce substantial cycle-to-cycle changes in the observed spectroscopic behavior.

## 3 Conclusions

In this work, we used time-resolved surface-enhanced Raman spectroscopy (TR-SERS) to resolve the dynamic evolution of nitrate-derived species during the NO_3_RR and NO_2_RR on silver under operating conditions. Correlation of spectroscopic signatures with electrochemical responses enabled the identification of vibrational fingerprints associated with aqueous and adsorbed nitrate species and revealed potential-dependent changes consistent with a redistribution between bridge- and chelate-adsorption geometries (*NO_3,bridge_ and *NO_3,chelate_, respectively). Resolving these adsorption dynamics in real time provides insights into the early stages of nitrate reduction, particularly the nitrate-to-nitrite transformation, which is widely proposed to constitute the rate-determining step in the NO_3_RR.

Complementary DFT calculations support the spectroscopic assignments and indicate the progressive weakening of nitrate and nitrite adsorption under increasingly reducing conditions. This overall tendency is retained across the terrace Ag(111) and stepped Ag(211) models despite quantitative differences in adsorption energetics and local binding geometries. Constant-potential calculations further show that surface charging can quantitatively modify the predicted stability ranges of nitrate- and nitrite-containing surface states. Increasing hydrogen coverage also alters the relative stability of the bridge and chelate nitrate configurations, bringing them close to energetic degeneracy at 0.25 ML H*. The calculated vibrational frequencies are additionally sensitive to hydrogen coverage, coadsorbed species, and local surface structure, highlighting the influence of the immediate interfacial environment on the observed spectroscopic signatures.

Together, the experimental and computational results provide a molecular-level picture of the potential-dependent adsorption and transformation of interfacial nitrate species under reaction conditions. The combination of time-resolved vibrational spectroscopy with atomistic modeling provides a framework for interrogating dynamic interfacial processes in NO_3_RR and other electrocatalytic reactions involving nitrogen-containing intermediates, including C–N coupling reactions. By tracking the evolution of surface species alongside the electrochemical response, this approach can help resolve potential-dependent changes in adsorption, local coordination, and reaction intermediates that are difficult to distinguish using static measurements alone. Ultimately, these insights provide a basis for establishing structure–reactivity relationships that can inform the development of more selective nitrogen-conversion electrocatalysts.

## Supplementary Material



## Data Availability

The computational data underlying this study are openly available in ioChem-BD at https://iochem-bd.bsc.es/browse/review-collection/100/492725/8d30d911111a480684d731de.

## Notes

Experimental methods and computational details (PDF)
