# Supplementary material for: Time-Resolved Raman Spectroscopy of Nitrate Electroreduction Reveals Dynamic Interfacial Adsorbate Evolution
Source: JACS Au. 2026 Oct 10;6(10):5785–97. doi: 10.1021/jacsau.6c01050 (PMC13714940; doi:10.1021/jacsau.6c01050)
Supplement: Supplementary file 1 [file au6c01050_si_001.pdf]

Supporting Information

Time-Resolved Raman Spectroscopy of Nitrate Electroreduction Reveals Dynamic Interfacial Adsorbate Evolution

Gabriel F. Costa,[1] Jaxiry S. Barroso-Martínez,[1] Dídac-Armand Fenoll,[2] Conor Brennan-Pollak,[3] Max García-Melchor,[2,3,4,*] María Escudero-Escribano[1,5,*]

[1]*Catalan Institute of Nanoscience and Nanotechnology (ICN2), CSIC and Barcelona Institute of Science and Technology, UAB Campus, 08193 Bellaterra, Barcelona, Spain*

[2]*CIC energiGUNE, Parque Tecnológico, C/Albert Einstein 48, 01510 Vitoria-Gasteiz, Spain*

[3]*School of Chemistry, Trinity College Dublin, Dublin 2, Ireland*

[4]*IKERBASQUE, Basque Foundation for Science, Plaza de Euskadi 5, 48009 Bilbao, Spain*

[5]*Catalan Institution for Research and Advanced Studies (ICREA), Pg. Lluís Companys 23, 08010 Barcelona, Spain*

* Corresponding authors: maxgarcia@cicenergigune.com ; maria.escudero@icn2.cat

Table of Contents

S1. Experimental Methods S2

S1.1. Electrochemical Methods for the Characterization of Silver for Nitrate and Nitrite Reduction S2

S1.2. Time-Resolved Surface-Enhanced Raman Spectroscopy S3

S2. Computational Details S10

S2.1. Computational Methodology. S10

S2.2. DFT Analysis of *H Adsorption on Flat and Defective Ag(111) Surfaces S12

S2.3. DFT Analysis $*NO_3$ and $*NO_2$ Adsorption on Flat Ag(111) Surfaces S14

S2.4. Computed Frequencies for $*NO_3$ and NO_2 Normal Modes on Flat and Defective Ag(111) Surfaces S18

S3. References S20

S1. Experimental Methods

S1.1. Electrochemical Methods for the Characterization of Silver for Nitrate and Nitrite Reduction

A silver rod electrode (Ag, 99.9%, 3.0 mm internal diameter) embedded in PEEK (6.0 mm external diameter; ALS, Japan) was used as the working electrode for cyclic voltammetry (CVs) and Raman measurements. The Ag disc was roughened by performing 5 consecutive CV cycles between –0.25 and 0.35 V vs the saturated calomel electrode (SCE), used as the reference electrode, in 0.1 M KCl.[1] A circular Ag wire was used as the counter electrode to ensure homogeneous roughening. After cycling, –0.35 V vs SCE was applied for 5 min to reduce any remaining surface oxides. **Figure S1** shows the potential–time profile of roughening procedure (a) and the corresponding fifth voltammogram (b). The roughened Ag electrode was used for SERS measurements to provide plasmonic enhancement of adsorbate signals and was also characterized electrochemically.

CVs of the roughened Ag electrode and a polycrystalline Ag disc (MaTeck, 99.9999% purity, 5 mm diameter) in hanging-meniscus configuration were recorded in a conventional four-neck glass cell equipped with a Luggin capillary to separate the reference electrode from the electrolyte solution. When the polycrystalline Ag electrode was used, the geometric area was employed to normalize the current. Ag surfaces were prepared by polishing on a microcloth (Struers) using diamond suspensions of 3, 1, and 0.25 µm (Struers) for 3 min at each step. The electrodes were then immersed in ultrapure water (>18.2 MΩ cm, Millipore Milli-Q) and sonicated for 5 min between polishing steps. All glassware was cleaned by soaking overnight in a potassium permanganate solution (1 g L^{-1} $KMnO_4$ dissolved in 0.5 M H_2SO_4), then rinsed by immersion in dilute piranha solution and boiled four times in ultrapure water. A freshly prepared solution, purged with Ar before and during the measurements, was used for each experiment. All salts used to prepare the solutions—potassium phosphate monobasic (KH_2PO_4, ≥99.0 % purity), potassium phosphate dibasic (K_2HPO_4, ≥98.0 % purity), potassium nitrate (KNO_3, ≥99.0 % purity) and (KNO_2, ≥99.0 % purity)—were purchased from Sigma-Aldrich Merck. An Ag wire (99.99% purity) and a commercial RHE electrode (Gaskatel) were used as counter electrode and reference electrode, respectively.

Lead underpotential deposition (Pb upd) was used to estimate the electrochemically active surface area (ECSA) of the roughened electrode (**Figure S2**). CVs for Pb upd were

recorded at 5 mV s^{-1} between –0.4 to –0.2 V vs SCE in a cell containing 0.1 M $KClO_4$ + 0.002 M $Pb(ClO_4)_2$. The Pb upd charge measured on flat Ag was used to determine the specific charge per unit area (260.77 μC cm^{-2}), which is in agreement with the literature.[2] Then, we estimated the ECSA of roughened Ag as the ratio between the measured charge for Pb upd on roughened Ag (red line in **Figure S2**) and the specific charge of Pb upd on Ag.

Scanning electron microscopy (SEM) images were acquired using XHRSEM Magellan 400L microscope, through-the-lens detector (TLD) and acceleration voltage of 5 kV. Two different magnifications for the samples analyzed were acquired: 50000 (**Figures S4a,c**) and 150000 (**Figures S4b,d**) with horizontal field width (HFW) of 5.97 and 1.99 μm, respectively.

Nitrite quantification (**Figure S5**) was performed using the colorimetric Griess method using UV-Vis spectrometer acquiring the spectra from 400 to 800 nm. The procedure involved combining 2.5 mL of the sample with 500 μL of Griess reagent (prepared from 50 mL of water, 2.94 mL of phosphoric acid >85%, 1 g of sulfanilamide, and 0.1 g of N-(1-naphthyl)ethylenediamine).

Electrolyses were carried out in an H-type cell with two compartments, separated by a Nafion 117 membrane that had been pretreated by soaking overnight in 0.5 mol L^{-1} H_2SO_4. The working electrode was flat or roughened Ag, a commercial reversible hydrogen electrode (RHE, Gaskatel) served as the reference, and a Pt wire placed in the anolyte compartment was used as the counter electrode.

S1.2. Time-Resolved Surface-Enhanced Raman Spectroscopy

TR-SERS measurements were performed using a Ventacon glass spectroelectrochemical cell with a three-electrode configuration consisting of roughened Ag as the working electrode, a commercial RHE (Gaskatel) as the reference electrode, and an Ag wire as the counter electrode. The electrolyte consisted of 0.1 M $K_xH_{3-x}PO_4$ (pH 7) containing 0.1 M KNO_3 or KNO_2. Spectra were collected using a Renishaw Raman InVia microscope during three consecutive CV cycles between 0.6 to –0.8 V vs RHE at a scan rate of 5 mV s^{-1}, with a 4 s acquisition time. Static spectra centered at 1200 cm^{-1} were acquired using a 532 nm laser at 50 mW. RamanSPy,[3] an open-source Python package, was used for data analysis, including heatmap generation and tracking of peak evolution.

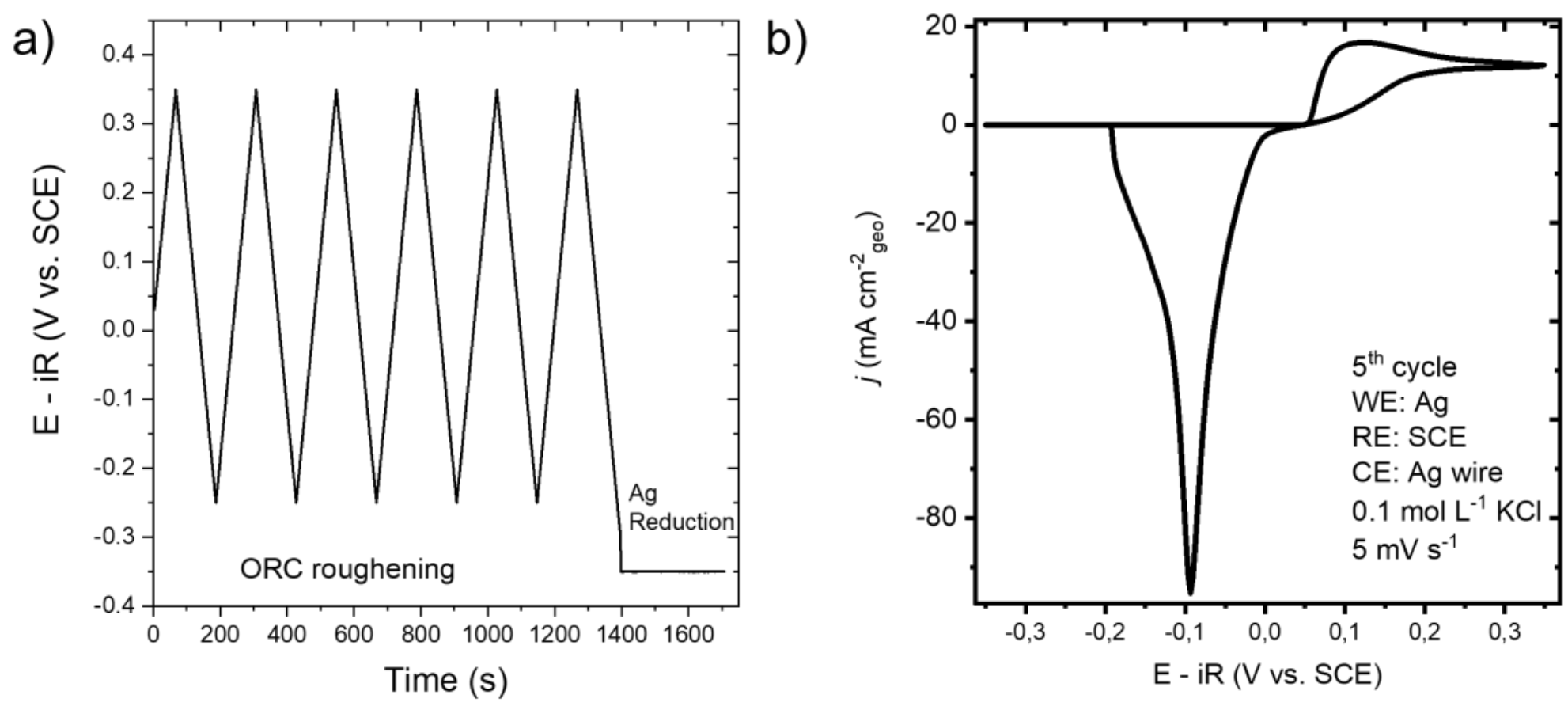

Figure S1. Electrochemical roughening of the Ag surface in 0.1 M KCl. (a) Potential–time profiles for five consecutive CV cycles used for surface roughening. (b) Voltammogram corresponding to the fifth cycle. Measurements were performed using an Ag rod as the working electrode, a circular Ag wire as the counter electrode, and a saturated calomel electrode (SCE) as the reference electrode at a scan rate of 5 mV s⁻¹.

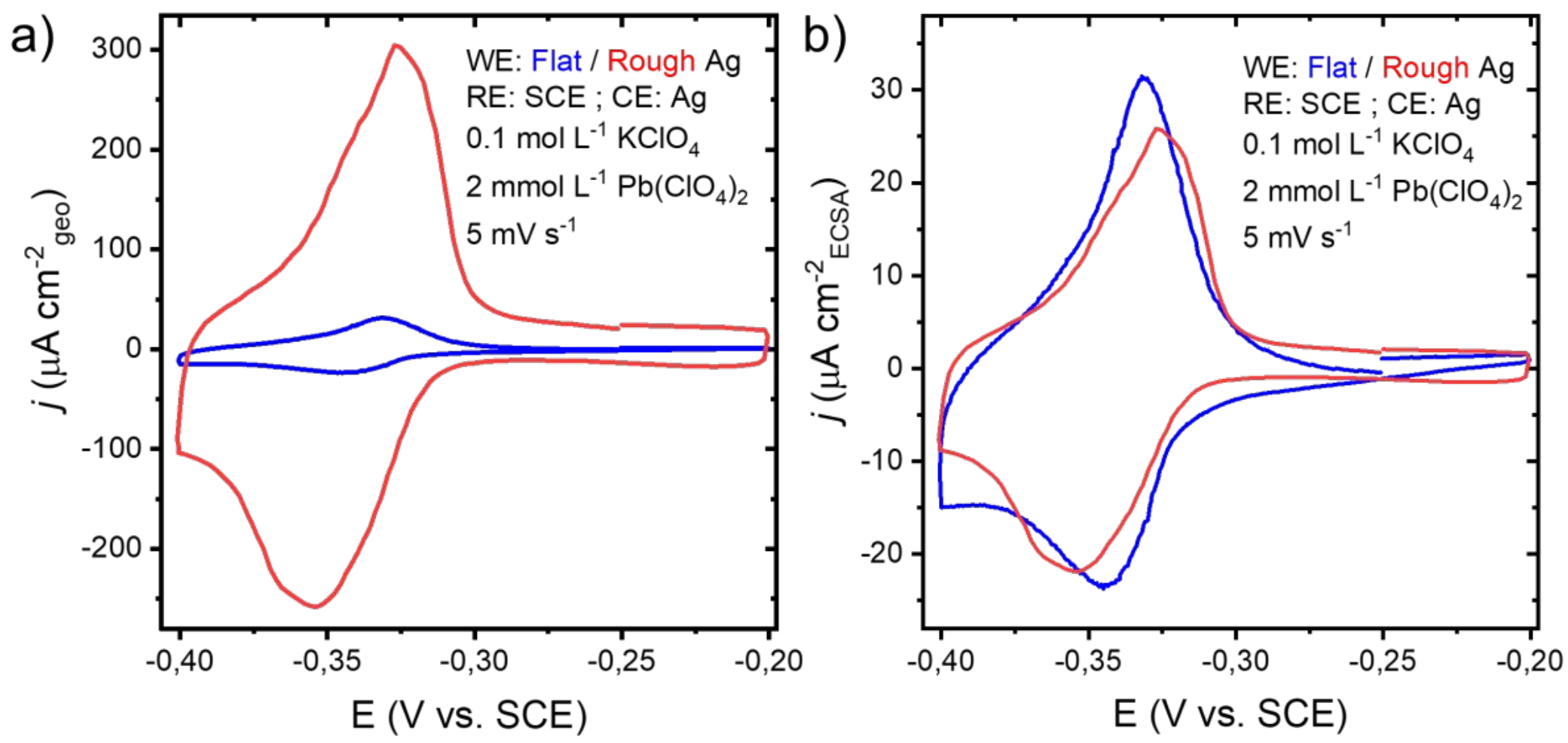

Figure S2. Cyclic voltammograms of Pb underpotential deposition (Pb upd) on a flat Ag disc (blue) and a roughened Ag electrode (red). Current densities are normalized by (a) the geometric surface area and (b) the electrochemically active surface area (ECSA). Measurements were performed using a saturated calomel electrode (SCE) as the reference electrode and an Ag wire as the counter electrode. CVs were recorded at a scan rate of 5 mV s⁻¹ between –0.40 to –0.20 V vs SCE in an electrolyte containing 0.1 M $KClO_4$ and 0.002 M $Pb(ClO_4)_2$.

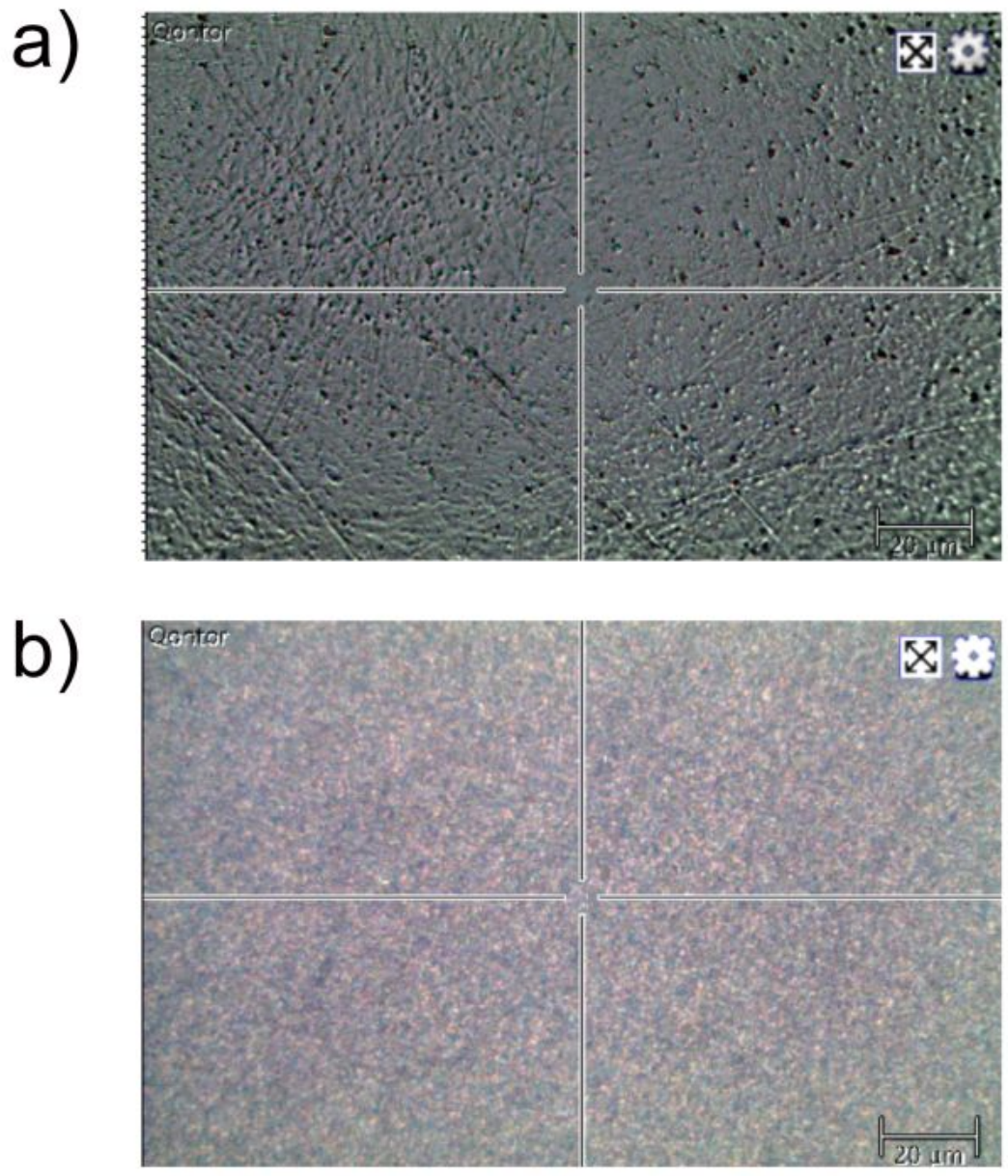

Figure S3. Optical micrographs of the Ag disc before (a) and after (b) electrochemical roughening.

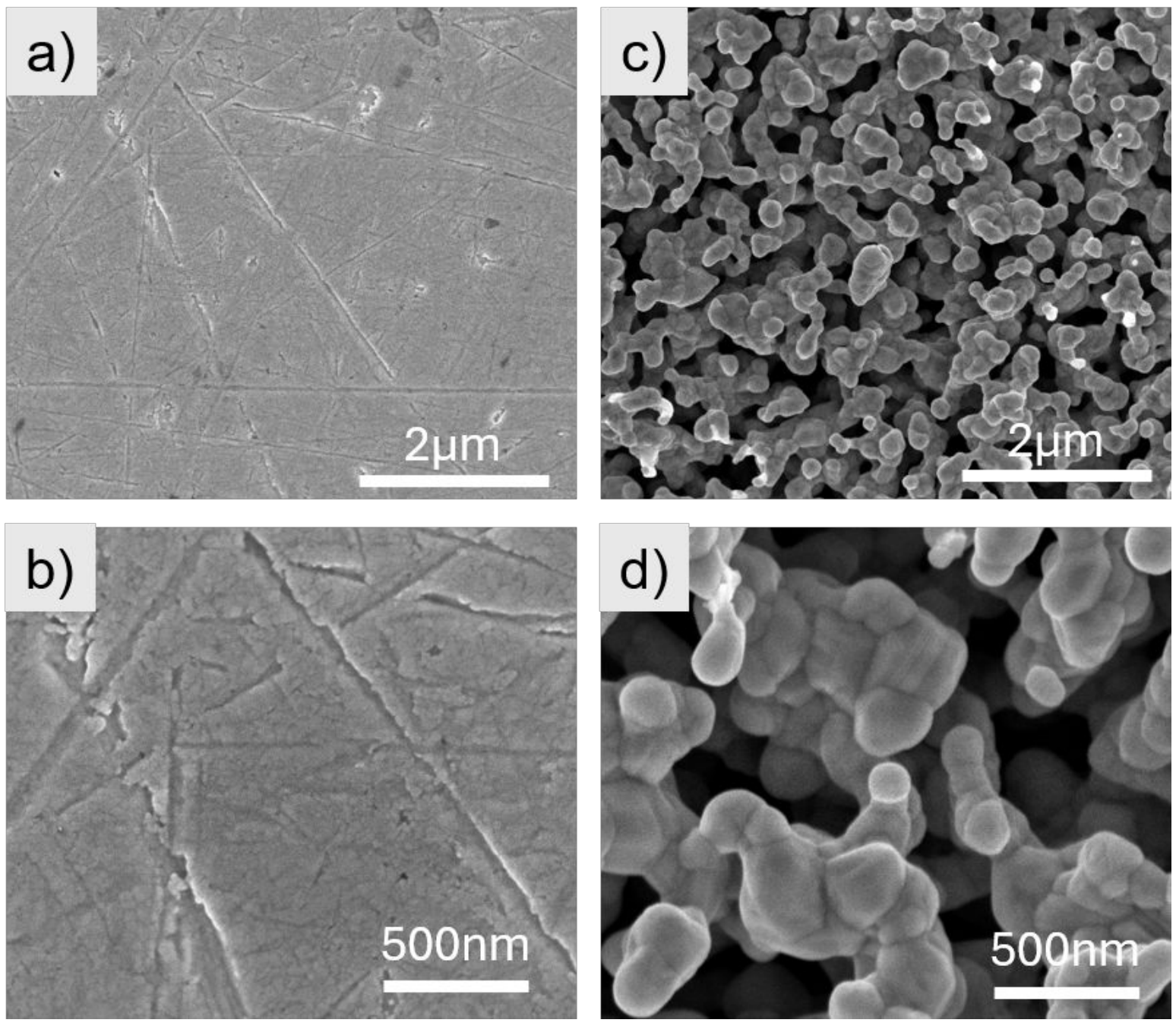

Figure S4. Scanning electric microscopy images of the Ag disc before (a,b) and after (c,d) electrochemical roughening.

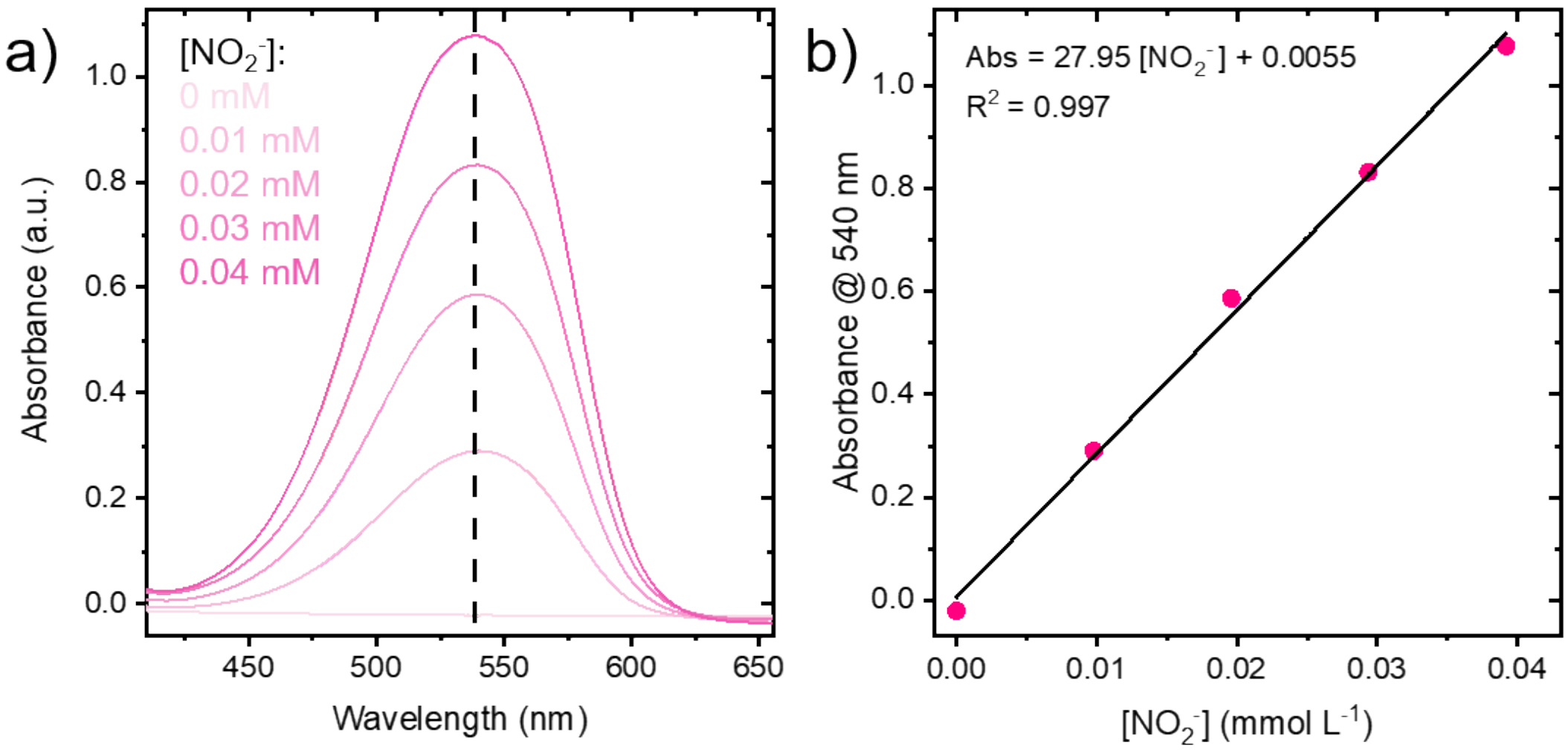

Figure S5. Calibration curve for nitrite quantification using Griess method. (a) UV-Vis spectra for solutions containing 0.01 – 0.04 mmol L^{-1} KNO_2. (b) Calibration curve considering the absorbance measured at 540 nm (dotted line in (a).

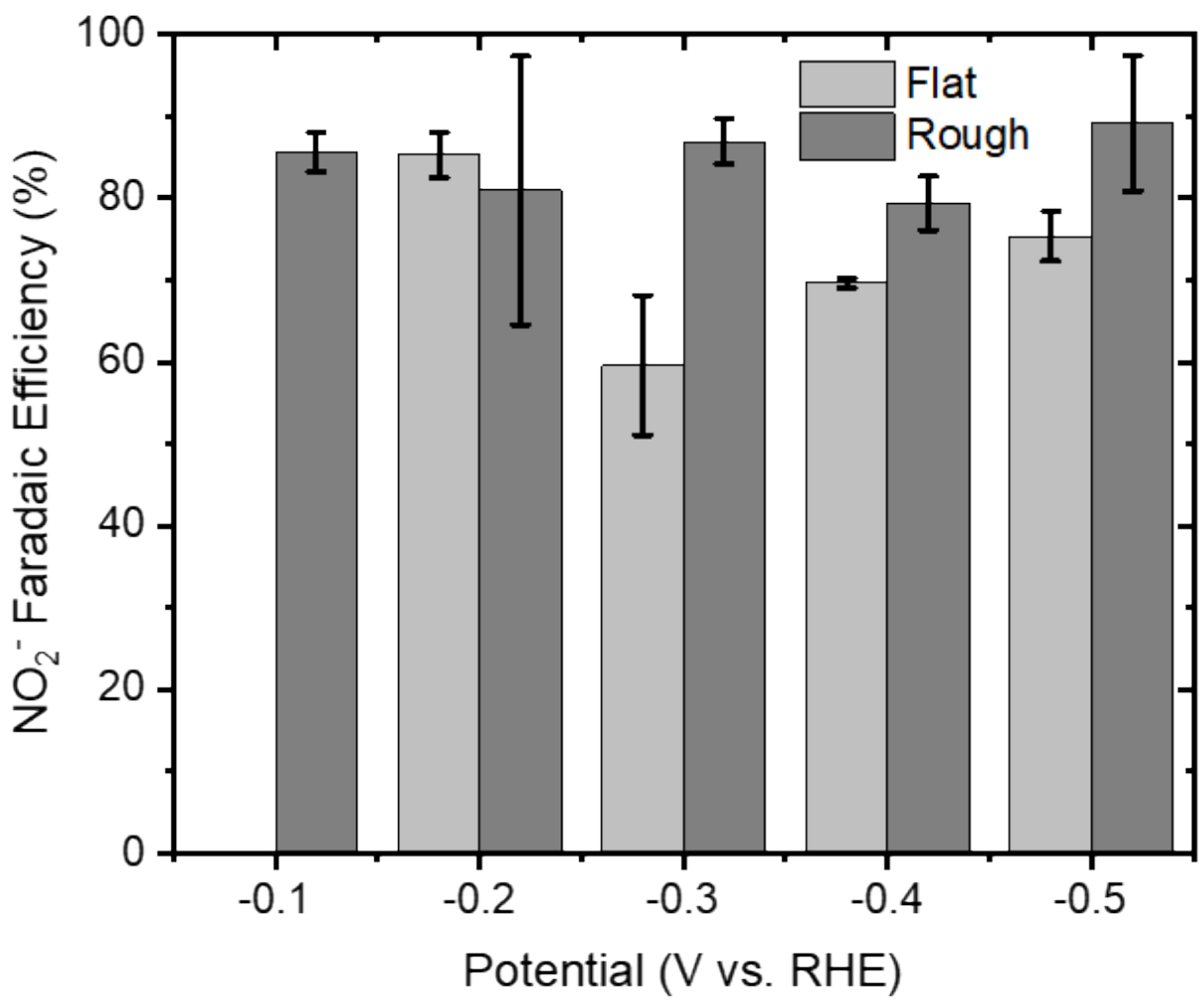

Figure S6. Faradaic efficiencies towards nitrite for flat (light gray) and roughened (dark gray) Ag obtained for 30-min electrolysis performed in a two-compartment cell separated by a Nafion 117 membrane using flat or roughened Ag as the working electrode, a reversible hydrogen electrode (RHE) as the reference electrode, and a Pt wire as the counter electrode. The catholyte contained 100 mM KNO_3. Potentials were applied for 30 min. The error bars denote the standard deviation of three independent electrolysis experiments were performed under each condition. All potentials were iR-compensated.

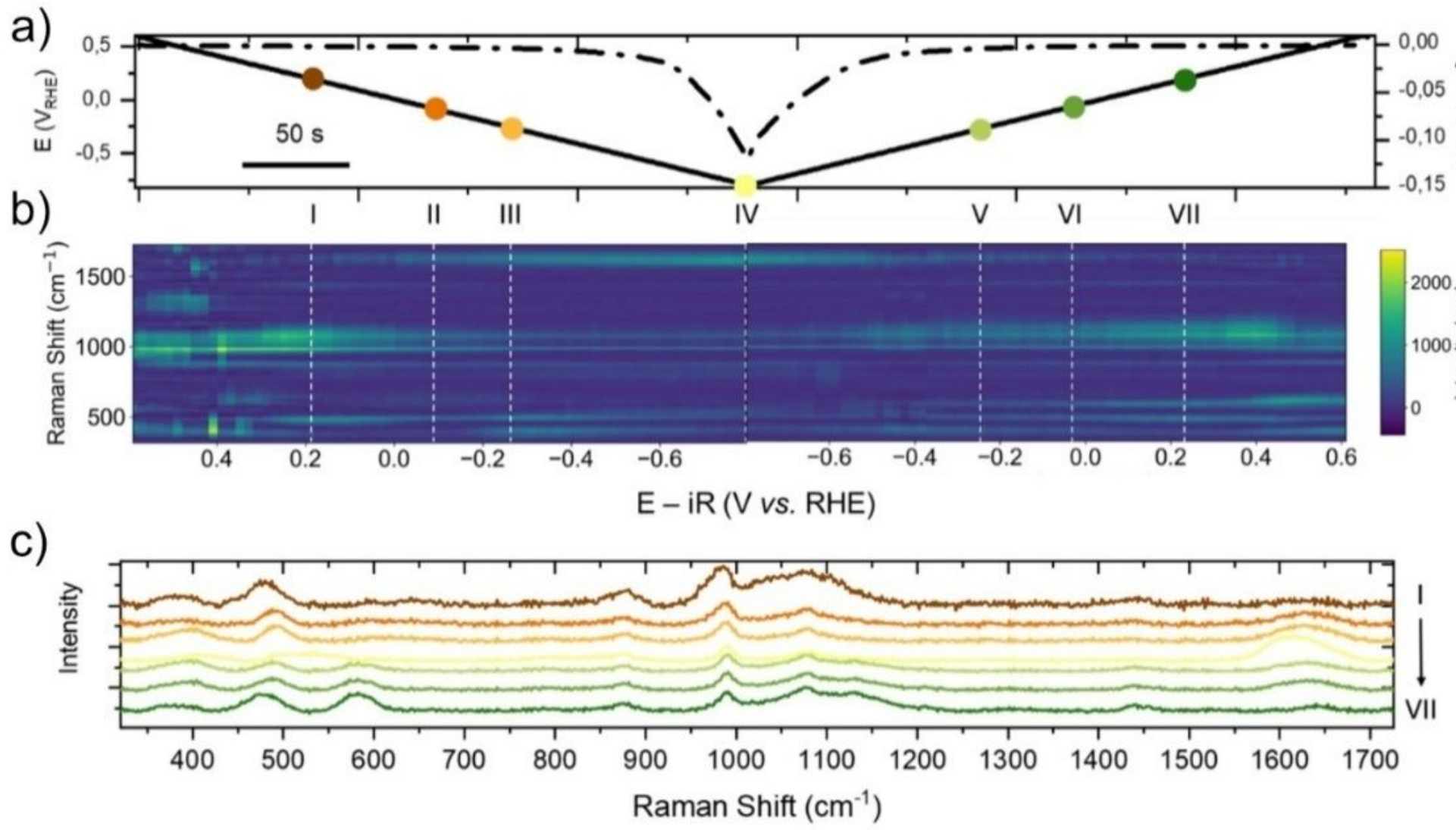

Figure S7. Time-resolved Raman measurements under blank electrolyte conditions. (a) Relationship between applied potential (black line, left axis) and current density (dotted line, right axis) during the third cycle of cyclic voltammetry. (b) Heatmap showing the evolution of the Raman spectra as a function of applied potential. Dotted white lines labeled I–VII indicate the spectra displayed in (c). (c) Raman spectra acquired at +0.20, –0.08, –0.25, and –0.80 V vs RHE during the cathodic scan and at –0.25, –0.08, and +0.20 V vs RHE during the anodic scan (spectra I–VII, respectively). Peaks of interest are highlighted with circles. CVs were recorded at a scan rate of 5 mV s^{-1} between +0.60 and –0.80 V vs RHE using roughened Ag as the working electrode, RHE as the reference electrode, and an Ag wire as the counter electrode in an electrolyte containing 0.1 M $K_xH_{3-x}PO_4$ (pH 7). Raman spectra were collected using a 532 nm laser (50 mW) with a 4 s acquisition time and a 50× objective.

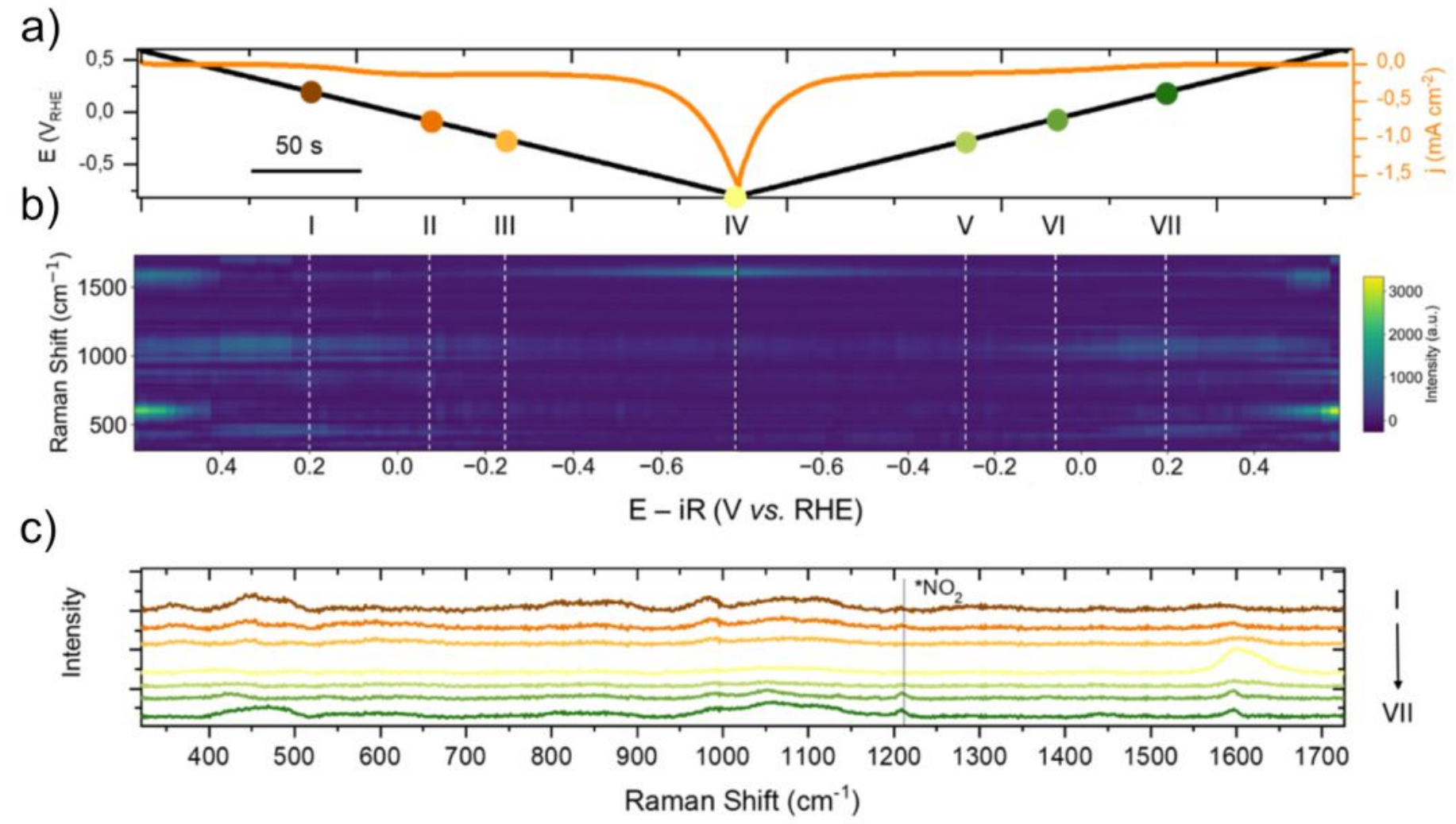

Figure S8. Time-resolved Raman measurements under nitrite reduction electrolyte conditions. (a) Relationship between applied potential (black line, left axis) and current density (dotted line, right axis) during the third cycle of cyclic voltammetry. (b) Heatmap showing the evolution of the Raman spectra as a function of applied potential. Dotted white lines labeled I–VII indicate the spectra displayed in (c). (c) Raman spectra acquired at +0.20, –0.08, –0.25, and –0.80 V vs RHE during the cathodic scan and at –0.25, –0.08, and +0.20 V vs RHE during the anodic scan (spectra I–VII, respectively). Peaks of interest are highlighted with circles. CVs were recorded at a scan rate of 5 mV s^{-1} between +0.60 and –0.80 V vs RHE using roughened Ag as the working electrode, RHE as the reference electrode, and an Ag wire as the counter electrode in an electrolyte containing 0.1 M $K_xH_{3-x}PO_4$ (pH 7) + 0.1 M KNO_2. Raman spectra were collected using a 532 nm laser (50 mW) with a 4 s acquisition time and a 50× objective lens.

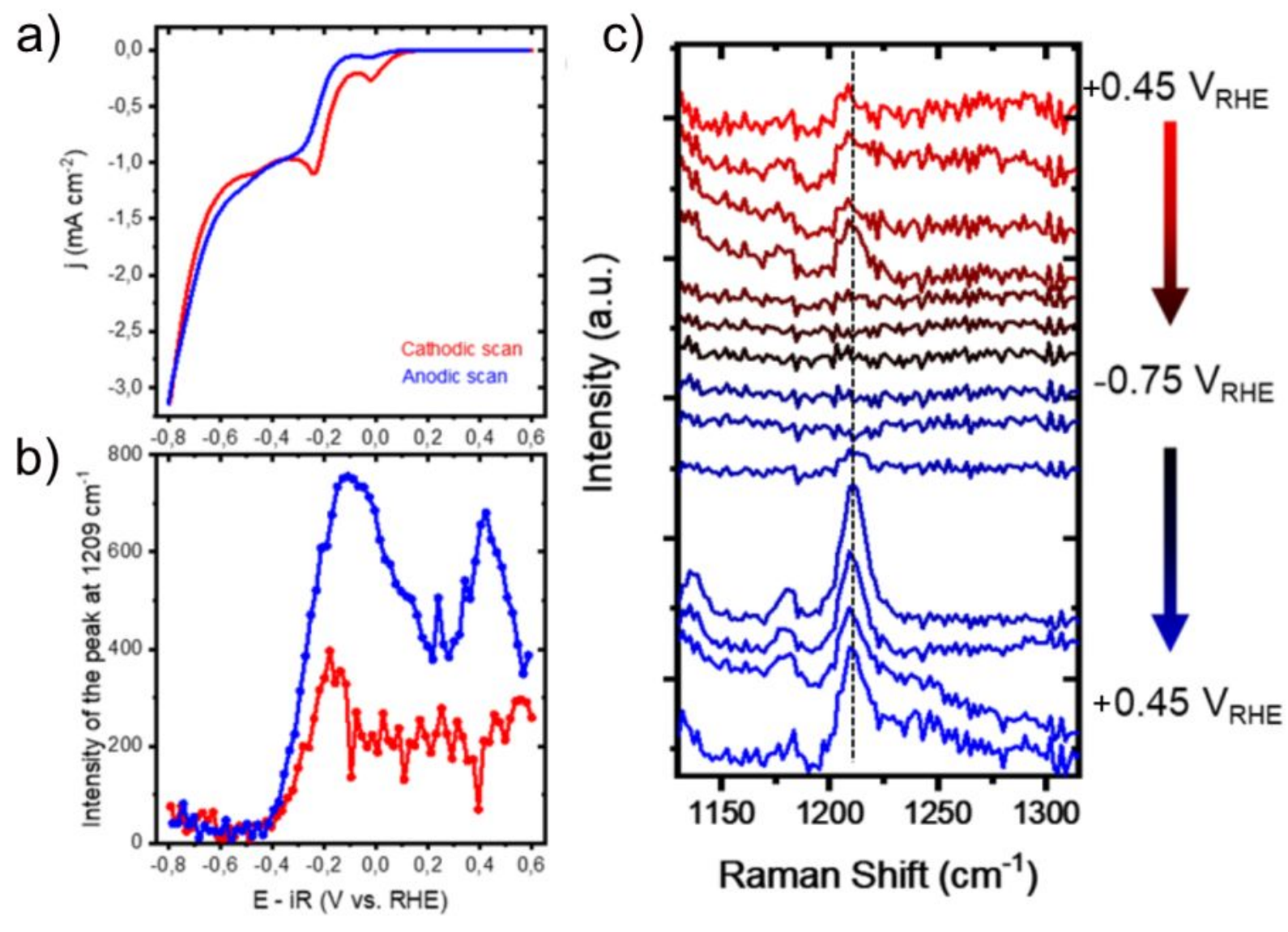

Figure S9. (a) Cyclic voltammogram corresponding to the third NO_3RR cycle on Ag. (b) Evolution of the intensity of the band centered at 1209 cm^{-1} as a function of applied potential for the voltammogram shown in (a). Cathodic and anodic scans are shown as red and blue traces, respectively. (c) Representative spectra acquired during the cathodic scan from +0.45 to –0.75 V vs RHE (shades of red) and during the anodic scan from –0.75 to +0.45 V vs RHE (shades of blue). CVs were recorded at 5 mV s^{-1} using roughened Ag as the working electrode, RHE as the reference electrode, and an Ag wire as the counter electrode in an electrolyte containing 0.1 M $K_xH_{3-x}PO_4$ (pH 7) and 0.1 M KNO_3. Raman spectra were collected using a 532 nm laser (50 mW) with a 4 s acquisition time and a 50× objective lens.

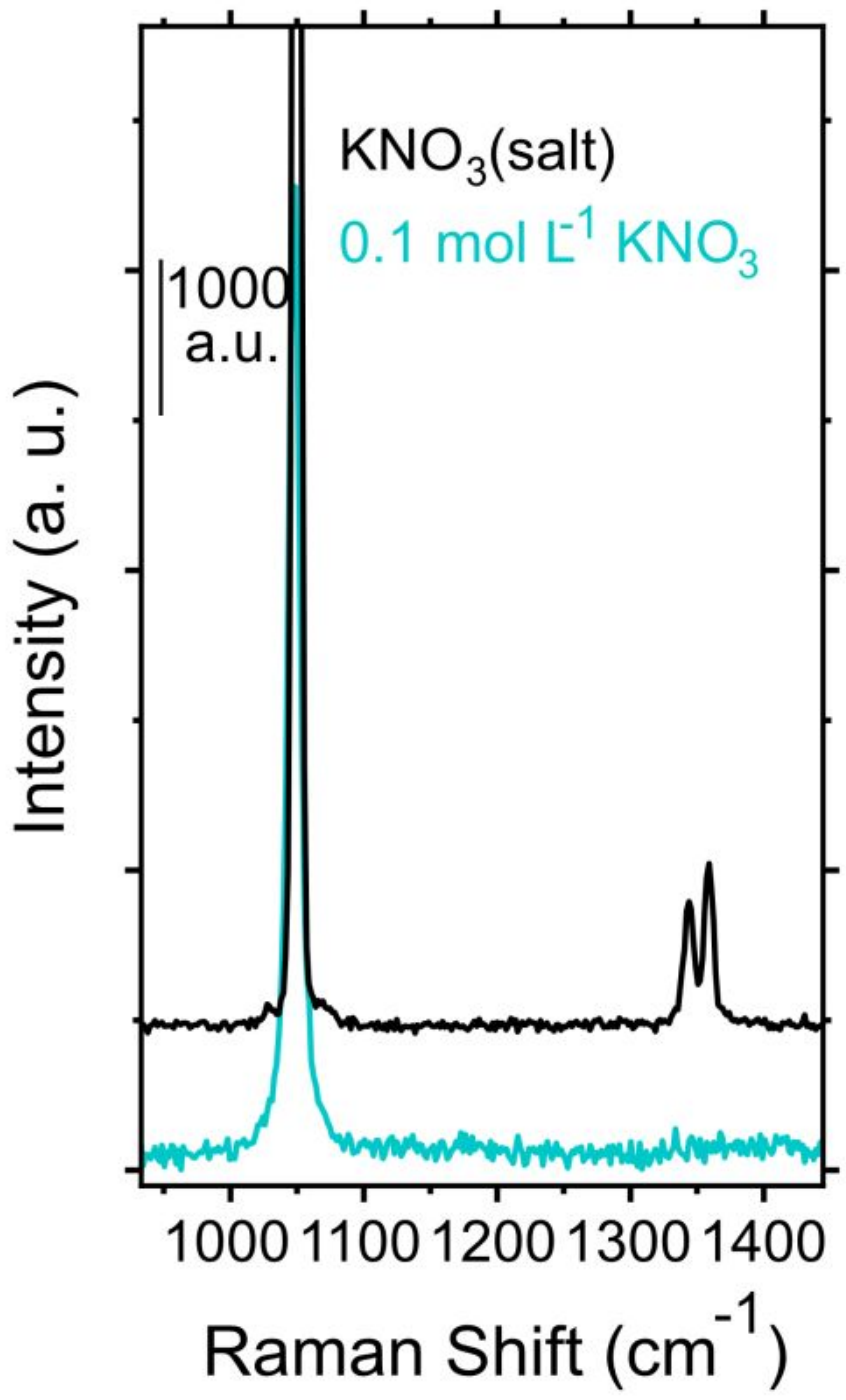

Figure S10. Raman spectra of solid (black) and aqueous (aqua blue) KNO_3. Raman spectra were collected using a 532 nm laser (50 mW) with a 4 s acquisition time and a 50× objective lens.

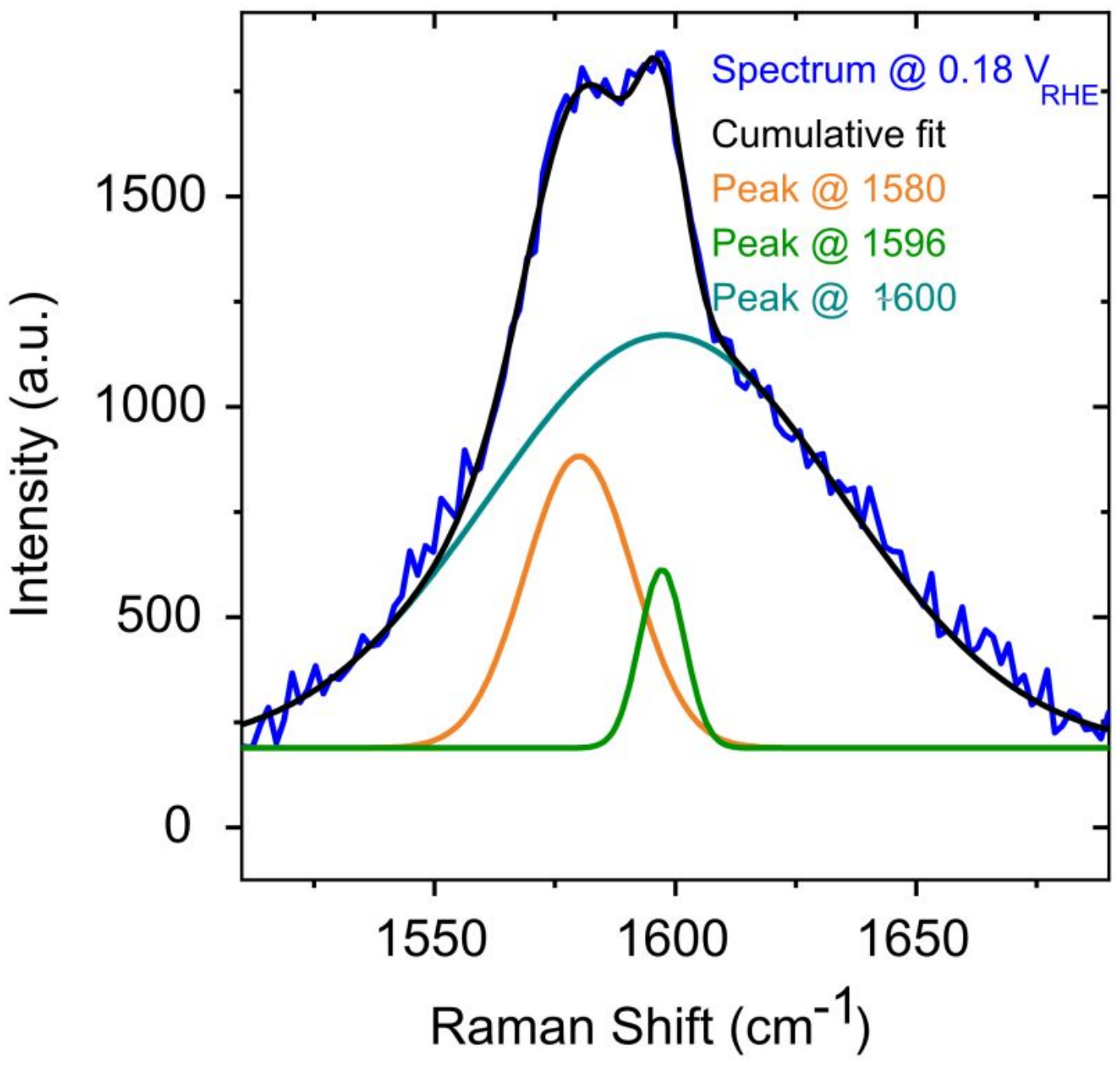

Figure S11. Gaussian fits of the peaks found in the spectrum acquired at +0.18 V vs RHE (dark blue trace). Deconvolution of the 1500–1700 cm^{-1} region yields peaks centered at 1580, 1596, and ~1600 cm^{-1} (orange, green, and aqua blue traces, respectively). The cumulative fit is shown in black.

S2. Computational Details

S2.1. Computational Methodology

Periodic, spin-polarized density functional theory (DFT) calculations were performed using the Vienna Ab Initio Simulation Package (VASP, version 6.4.2).[4–7] The Bayesian error estimation functional with van der Waals dispersion (BEEF-vdW) was used to describe exchange-correlation effects.[8–10] Methodological parameters were selected based on previous theoretical studies of nitrate electroreduction and related electrochemical processes.[11–13] Valence electrons were represented using plane waves with a kinetic energy cut-off of 500 eV, while core electrons were described using projector-augmented wave (PAW) pseudopotentials.[14]

Electronic and ionic convergence thresholds were set to 10^{-6} eV and 0.01 eV $Å^{-1}$, respectively. Structure relaxations were performed using the conjugate-gradient algorithm with an ionic step size of 0.1 Å. Electronic occupancies were described using a Gaussian smearing of width 0.05 eV for gas-phase molecules and first-order Methfessel-Paxton smearing with width 0.2 eV for periodic surfaces.[15]

All structures were generated using the Atomic Simulation Environment (ASE) software.[16] The Ag(111) surface used in this work was previously modeled in related studies. Briefly, a *p*(4×4) surface supercell was cleaved from a bulk face-centered cubic (*fcc*) Ag obtained from the Materials Project database (ID: mp-81) and subsequently optimized using a Birch-Murnaghan equation-of-state fitting.[17,18] This procedure yielded an equilibrium lattice parameter of 4.22 Å.

Gas-phase molecules were optimized at the Γ-point in cubic cells containing 15 Å of vacuum to minimize interactions between periodic images. For periodic slabs, a Γ-centred k-point grid corresponding to a density of approximately 6.12 ± 0.54 points $Å^{-1}$ was used to sample the Brillouin zone. A vacuum region of 15 Å was introduced along the direction perpendicular to the surface to avoid spurious slab–slab interactions. The flat Ag(111) slab consisted of four atomic layers, with the bottom two layers fixed at their bulk positions during structure optimization.

To model the roughened Ag surface, two defective surface models were constructed: an Ag adatom located at an *fcc* site (Ag_1) and a three-atom Ag aggregate occupying contiguous *fcc* sites (Ag_3). As for the flat slab, the two bottom layers of the defective slabs were kept fixed during geometry optimization.

Vibrational frequency calculations were performed using the finite-difference method with atomic displacements of ±0.02 Å. For gas-phase molecules, all atoms were displaced, whereas for surface calculations only the adsorbates (*H, $*NO_3$ and $*NO_2$ species) were vibrated. Gibbs energy corrections were calculated at 298.15 K and 1 atm using the VASPkit implementation of the ideal-gas model for gas-phase species and the harmonic approximation for adsorbate–surface structures.[19] Low-frequency spurious modes were replaced by real frequencies of 12 cm^{-1} following the approach proposed by Nørskov and coworkers.[20,21] The Gibbs energy of a system is herein defined according to **Equation 1**:

$$G = E - T{\cdot}S + (1)$$

Where T is the temperature and E, S and ZPE are the potential energy, the vibrational entropy and the zero-point energy of the system, respectively.

Adsorption Gibbs energies for the neutral *H, $*NO_3$, and $*NO_2$ species were calculated within the computational hydrogen electrode (CHE) framework at 0 V vs RHE (0_{RHE}) using H_2, HNO_3, and HNO_2 as reference molecules. The adsorption Gibbs energies were computed according to **Equations 2-4**:

$$\Delta G_{*nH}(0_{RHE}) = G_{*nH} - \left(G_{*} + \frac{n}{2}G_{H_2}\right)(2)$$

$$\Delta G_{*NO_3}(0_{RHE}) = G_{*NO_3} - \left(G_{*nH} + G_{HNO_3} - \frac{1}{2}G_{H_2}\right)(3)$$

$$\Delta G_{*NO_2}(0_{RHE}) = G_{*NO_2} - \left(G_{*nH} + G_{HNO_2} - \frac{1}{2}G_{H_2}\right)(4)$$

where G_{*NO_3}, and G_{*NO_2} correspond to the Gibbs energies of the adsorbed nitrogenous species, G_{*nH} is the Gibbs energy of the corresponding bare or partially H-passivated surface (n = 0, 4, 8, 12, or 16), and G_{H_2}, G_{HNO_3}, and G_{HNO_2} are the Gibbs energies of the reference molecules. The additional $\frac{1}{2}G_{H_2}$ term in Equations 3-4 accounts for the additional H atom introduced from the HNO_2 and HNO_3 references used.

The dependence of adsorption Gibbs energies on the applied potential, U_{RHE}, was introduced according to the CHE framework, **Equations 5-7**:

$$\Delta G_{*nH}(U_{RHE}) = \Delta G_{*nH}(0_{RHE}) + neU_{RHE} (5)$$

$$\Delta G_{*NO_3}(U_{RHE}) = \Delta G_{*NO_3}(0_{RHE}) - eU_{RHE} (6)$$

$$\Delta G_{*NO_2}(U_{RHE}) = \Delta G_{*NO_2}(0_{RHE}) - eU_{RHE} (7)$$

For non-bare surfaces, the potential-dependent correction arises solely from the $*NO_3$ and $*NO_2$ species because the corresponding H-covered surface is taken as the reference state.

Raman spectra were computed using Γ-point phonons obtained from finite-displacement calculations in VASP combined with the macroscopic dielectric tensor calculated through density functional perturbation theory. Off-resonance Raman activities were then obtained from derivatives of the polarizability tensor using a Python-based implementation.[22]

S2.2. DFT Analysis of *H Adsorption on Flat and Defective Ag(111) Surfaces

To rationalize the potential-dependent adsorption behavior observed experimentally, periodic DFT calculations were used to investigate the adsorption of *H, $*NO_3$, and $*NO_2$ on flat and defective Ag(111) surfaces. The predicted resting states of Ag(111) in the presence of *H alone are discussed first, whereas adsorption of $*NO_3$ and $*NO_2$ is addressed in **Section S2.3**.

For flat Ag(111), hydrogen adsorption is weak even at the most favorable *fcc* sites, resulting in a relatively cathodic onset for the formation of a full *H monolayer (1.0 ML H) (**Figure S12a**), in agreement with previous studies.[12] At –0.60 V vs RHE, only one quarter of the *fcc* sites are predicted to be occupied, whereas the remaining *fcc* sites become populated at approximately –0.80 V vs RHE. These coverage transitions are not expected to be abrupt, as the calculated stability lines are separated only by small Gibbs energy differences.

Negligible differences are expected on the Ag_1 model, as the adatom-generated sites are less stable and the distant terrace *fcc* sites are preferentially populated regardless of the defect. In contrast, the surface morphology of the Ag_3 model differs substantially from that of the flat Ag(111) surface. In this system, three terrace *fcc* sites are occupied by the Ag adatoms, and the remaining adsorption sites lie within one lattice spacing of the Ag aggregate. Adsorption of a single H atom on the available sites on Ag_3, which includes a three-coordinated hollow site and step-like sites associated with the aggregate, indicates

that terrace *fcc* sites remain the most favorable adsorption sites, although H binding is weaker than on flat Ag(111).

Based on the surface coverage analysis for Ag_3 (**Figure S12b**), the formation of 0.25 ML H coverage occurs at potentials approximately 0.10 V more negative than on flat Ag(111) (**Figure S12a**). This computed 0.10 V delay in the formation of 0.25 ML H on Ag_3 is consistent with the approximately 0.10 V more cathodic onset of the HER observed experimentally on the roughened Ag electrode. Furthermore, an *fcc* coverage of 0.06 ML H (corresponding to one *H adsorbate in the Ag_3 model) appears at slightly less cathodic potentials than the 0.25 ML H coverage predicted for the flat surface.

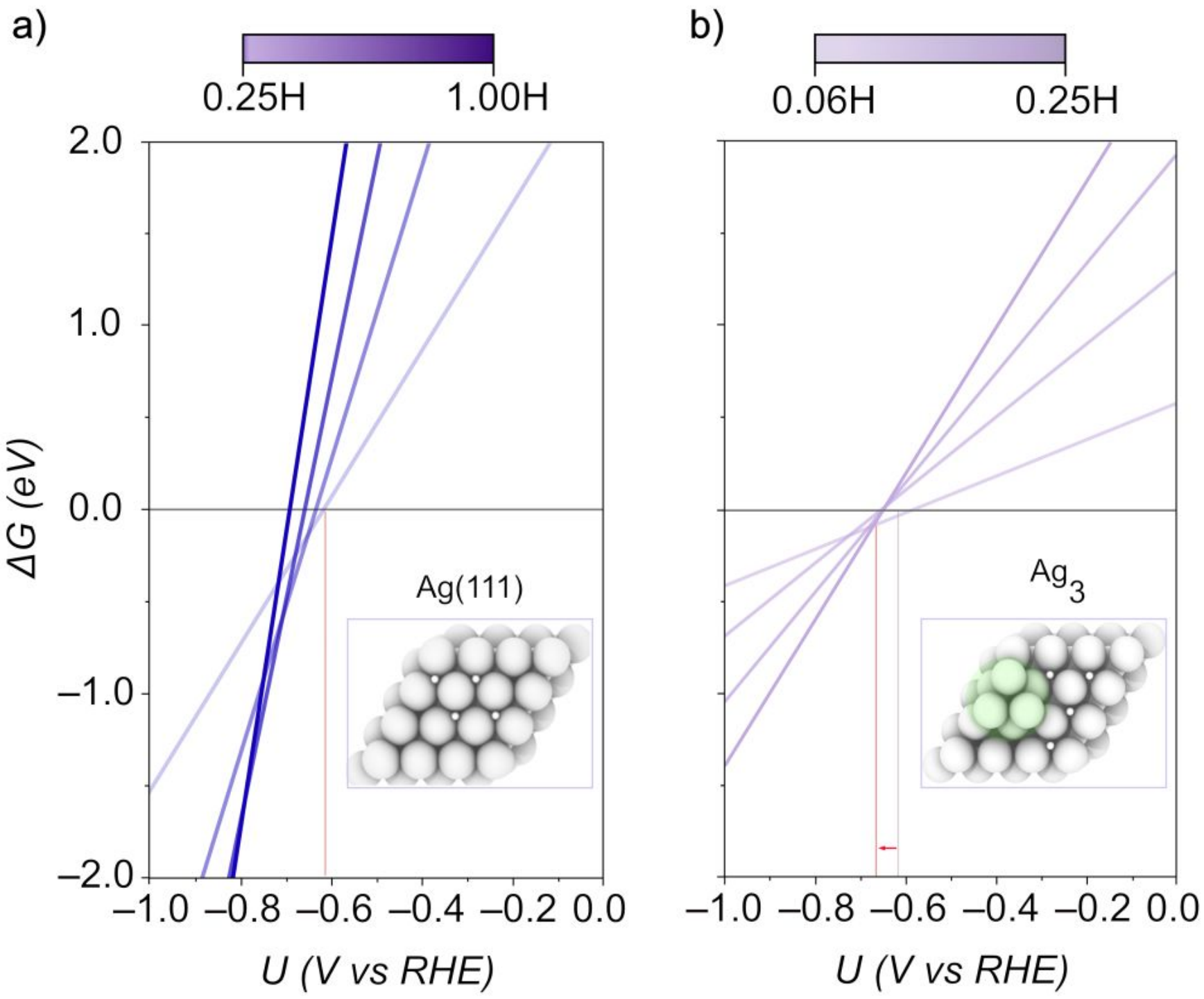

Figure S12. Surface hydrogen coverage analysis for Ag electrodes. Computed potential-dependent hydrogen surface coverage for (a) flat Ag(111) and (b) defective Ag_3. The color gradient indicates increasing *H coverage of terrace *fcc* sites. Insets show the corresponding 0.25 ML H coverages for flat Ag(111) and Ag_3. Red lines mark the onset potentials for the formation of the 0.25 ML H coverage. The red arrow in (b) highlights the potential difference between 0.06 ML H and 0.25 ML H coverages on Ag_3.

S2.3. DFT Analysis of $^*NO_3$ and $^*NO_2$ Adsorption on Flat Ag(111) and Stepped Ag(211). Selected exploration of $^*NO_3$ and $^*NO_2$ Adsorption on Ag(111) using the Constant Potential formalism

The adsorption modes considered for $^*NO_{3,X}$, where X = *bridge, chelate, fcc,* or *flat* (**Figure 4e)**, together with $^*NO_{2,bridge}$, are discussed in this section. Considering the potential range explored experimentally during the cathodic sweeps, the bare surface and the 0.25 ML H surface are proposed to represent the most relevant resting states of the Ag electrode. Nevertheless, higher hydrogen coverages were also examined to illustrate the progressive weakening of $^*NO_{3,bridge}$ (**Figure S13a**) and $^*NO_{2,bridge}$ (**Figure S13b**) at increasingly cathodic potentials.

Our calculations indicate that both nitrate and nitrite bind weakly to the bare Ag surface. For the bidentate *bridge* configuration at 0 V vs RHE, adsorption energies of $\Delta G_{*NO_{3,bridge}} =$ –0.06 eV and $\Delta G_{*NO_{2,bridge}} =$ –0.01 eV were obtained. Adsorption on surfaces with increasing hydrogen coverage, corresponding to progressively more cathodic conditions, was modeled by placing H atoms on terrace *fcc* sites while maximizing interactions with nitrogen-containing adsorbates.

At 0.25 ML H coverage, adsorption becomes thermodynamically unfavorable for both species. For example, adsorption energies of $\Delta G_{*NO_{3,bridge}} = +0.85$ eV and $\Delta G_{*NO_{2,bridge}} = +1.10$ eV are obtained as early as –0.30 V vs RHE. These results support the experimentally observed attenuation of nitrate- and nitrite-related Raman signals at increasingly cathodic potentials.

Increasing hydrogen coverage also affects the relative stability of nitrate binding geometries. As terrace *fcc* sites become progressively occupied by H atoms, $^*NO_{3,bridge}$ and $^*NO_{3,chelate}$ grow closer in stability. In particular, hydrogen-bonding interactions are proposed to stabilize the $^*NO_{3,chelate}$ configuration, which is otherwise less favorable on the bare surface. This behavior is consistent with the experimentally observed increase in the TR-SERS signal associated with $^*NO_{3,chelate}$ at negative potentials together with the relative weakening of the $^*NO_{3,bridge}$ mode intensity.

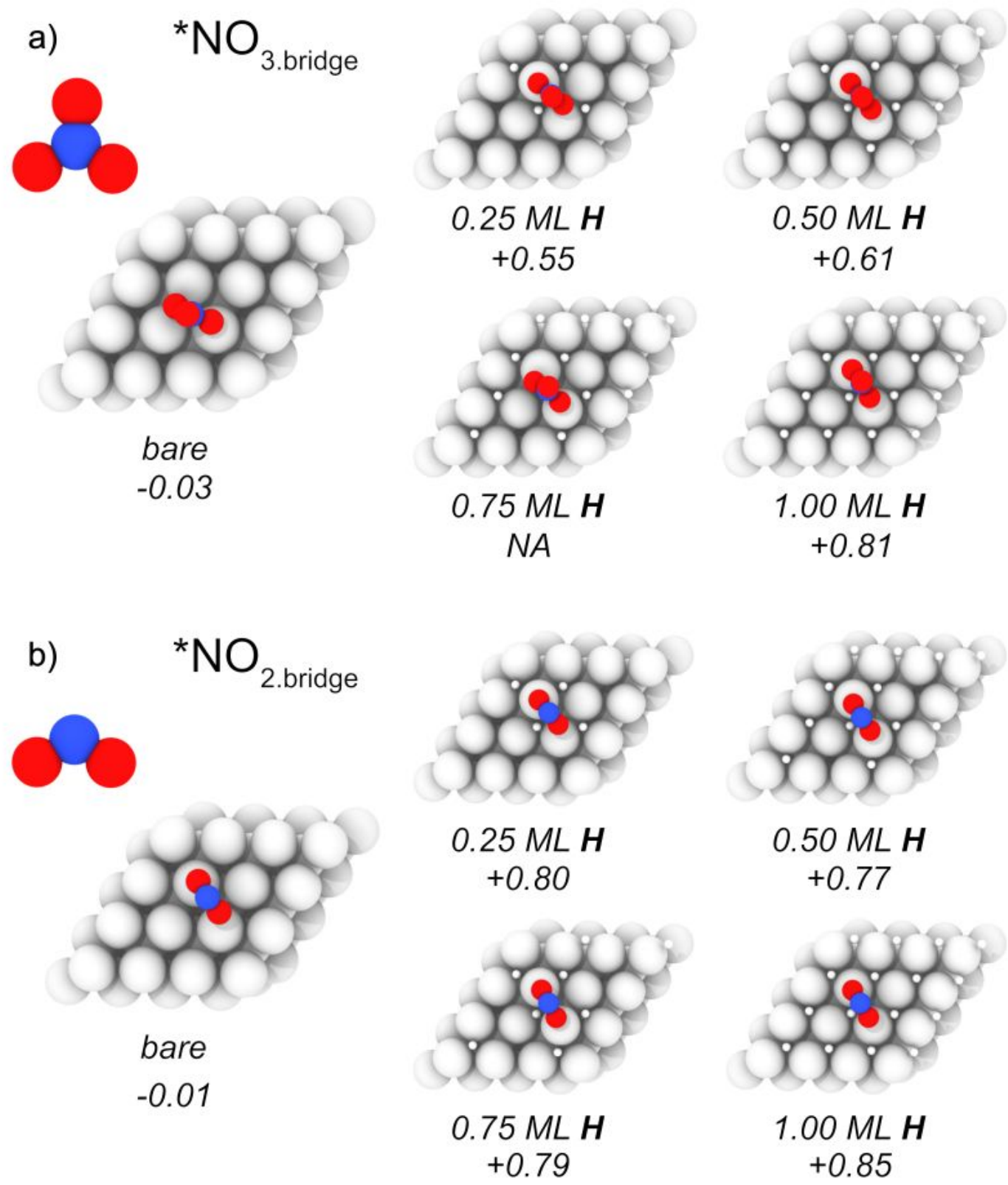

Figure S13. Resting states of flat Ag(111) with $*NO_x$ and *H co-adsorbates. Relaxed adsorption geometries of (a) nitrate and (b) nitrite on flat Ag(111) with increasing fractions of an *fcc* hydrogen monolayer. Gas-phase, Gibbs adsorption energies at 0 V vs RHE, in eV.

Adsorbate-induced surface reconstruction becomes more pronounced with increasing hydrogen coverage. On the bare surface, reconstruction is limited primarily to a slight upward displacement of the Ag atom coordinating the adsorbate. Importantly, no minimum energy could be found for nitrate adsorption for 0.75 ML H coverage (based on the frequency analysis), as one neighboring *H migrates toward an interstitial position upon adsorption of $*NO_{3,bridge}$ **(Figure S13)**. Reconstruction effects are largely suppressed on the fully hydrogen-covered surface. Equivalent trends are observed for nitrite adsorption, although surface reconstruction appears lower in extent. At 1.00 ML H coverage, explored configurations other than those shown in **Figure S13a,b** result in the evolution of HNO_3 or HNO_2 species.

To evaluate potential-dependent surface-charging effects beyond the conventional constant-charge computational hydrogen electrode (CC/CHE) treatment, we compared CC/CHE and constant-potential (CP) adsorption Gibbs energies for selected Ag(111) surface states (**Figure S14**). These include 0.25 ML *H, $*NO_3$ and $*NO_2$ on bare Ag(111), and $*NO_3$ and $*NO_2$ co-adsorbed with 0.25 ML *H. CP calculations were performed using a grand-canonical charge-interpolation scheme.[23] The CC/CHE and CP treatments yield closely comparable adsorption energetics for 0.25 ML *H, consistent with our previous systematic benchmark of *H adsorption on Ag(111).[13] In contrast, appreciable potential-dependent adsorption energetics of the nitrate- and nitrite-containing states differ appreciably between CC/CHE and CP treatments, resulting in quantitative changes in their predicted stability ranges.

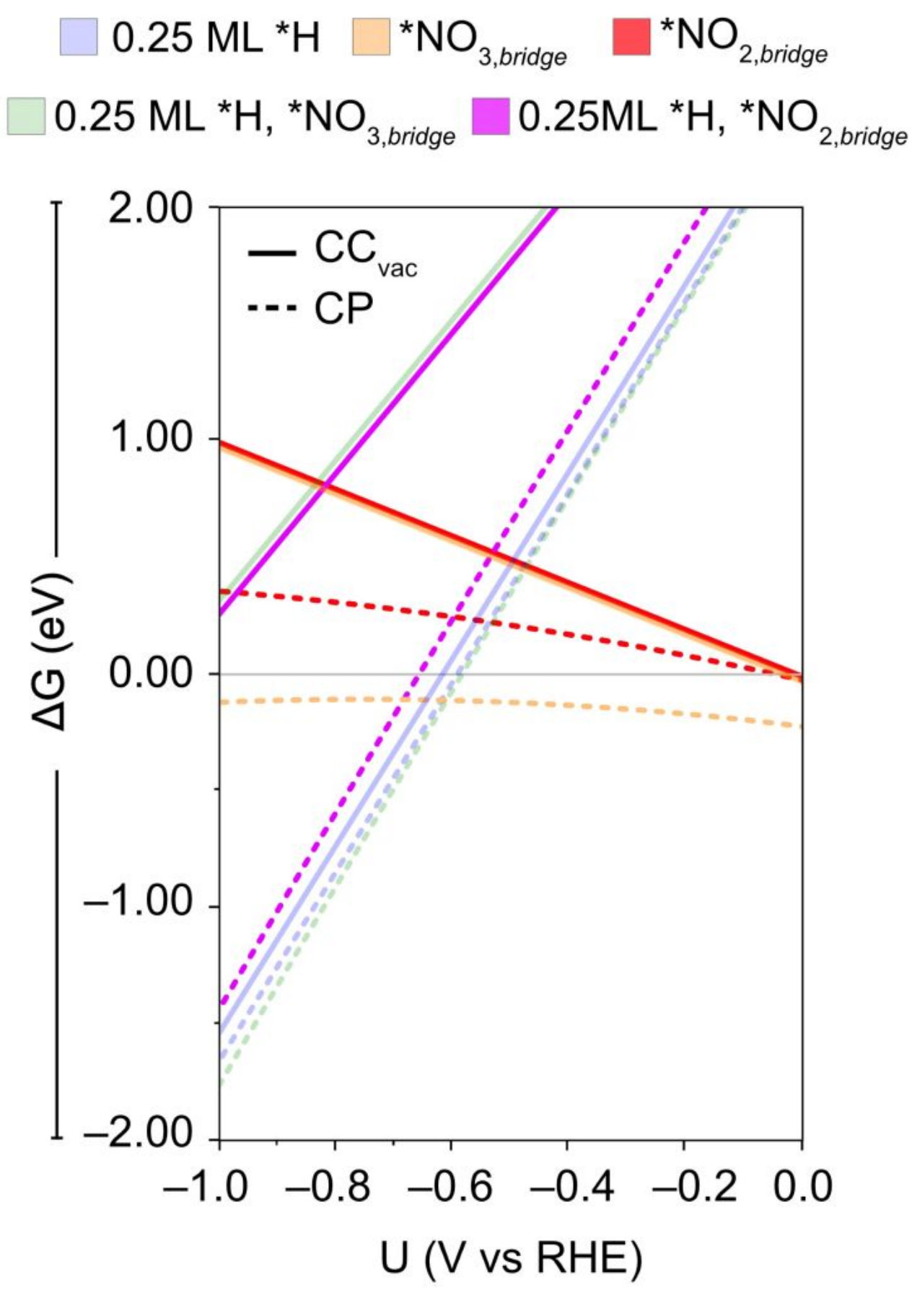

Figure S14. Comparison of constant-charge and constant-potential adsorption energetics on Ag(111). Potential-dependent adsorption Gibbs energies calculated using the constant-charge computational hydrogen electrode approach in the gas-phase (CC/CHE, solid lines) and the grand-canonical constant-potential approach (CP, dashed lines) for 0.25ML *H (blue), $*NO_{3,bridge}$ (orange) and $*NO_{2,bridge}$ (red) on bare Ag(111), and $*NO_{3,bridge}$ and $*NO_{2,bridge}$ co-adsorbed with 0.25ML *H (green and purple,

respectively). CP energies were obtained using the charge-interpolation scheme described in Ref. [23].

To complement the Ag(111) and localized defect models (Ag_1 and Ag_3), we additionally considered a stepped Ag(211) surface as a more extended representation of undercoordinated surface sites. The potential-dependent *H surface coverage was evaluated (**Figure S15a**), together with the adsorption energetics and geometries of $*NO_3$ and $*NO_2$ on bare and H-covered Ag(211) (**Figures S15b,c**).

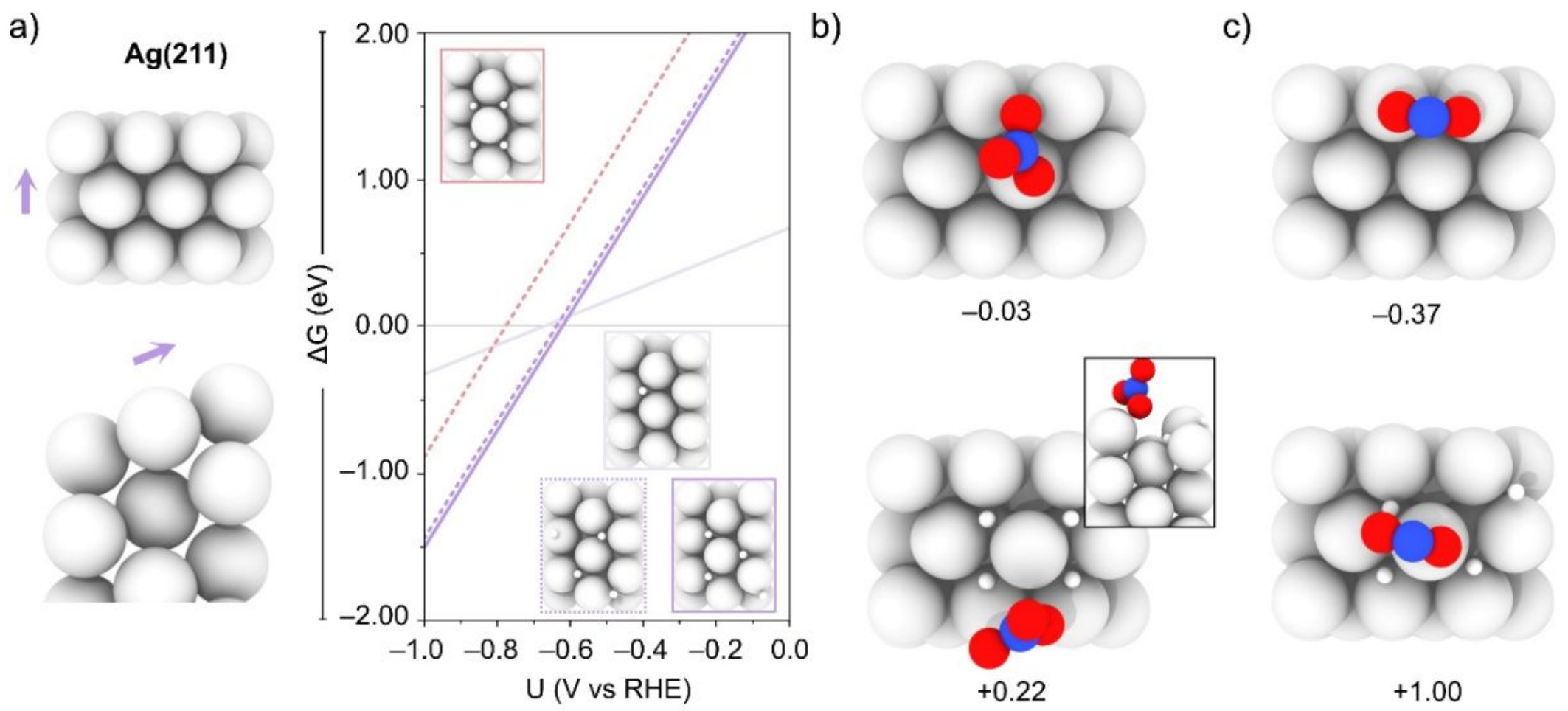

Figure S15. Surface states of *H, $*NO_3$, $*NO_2$ on stepped Ag(211). (a) Surface-coverage analysis for *H on Ag(211). Multiple configurations at the same H coverage were considered, with the corresponding optimized geometries color-coded in the inset. Relaxed adsorption geometries and gas-phase adsorption Gibbs energies at 0 V vs RHE for (b) $*NO_3$ and (c) $*NO_2$ on bare Ag(211) and Ag(211) with four co-adsorbed *H species. Adsorption Gibbs energies are given in eV.

The corresponding vibrational frequencies are summarized for the structures shown in **Figure S15** are presented in **Table S1:**

Table S1. Calculated vibrational frequencies (cm^{-1}) for selected normal modes of $*NO_3$ and $*NO_2$ adsorbed on bare Ag(211) (**Figures S15b** and **S15c**, respectively). The corresponding frequencies calculated on Ag(111) are given in parentheses.

	$\overline{\nu_3}$	$\overline{\nu_2}$	$\overline{\nu_1}$
$*NO_3$	1486<br>(1470)	1132<br>(1128)	957<br>(973)
$*NO_2$	1270<br>(1270)	1209<br>(1205)	779<br>(776)

S2.4. Computed Frequencies for $*NO_3$ and $*NO_2$ Normal Modes on Flat and Defective Ag(111), and on Stepped Ag(211)

To support the spectroscopic data and interpretation, off-resonance Raman activities were calculated for $*NO_{3,bridge}$ (**Figure S16a**) and $*NO_{2,bridge}$ (**Figure S16b**) on the bare Ag(111) surface. Discrepancies in the proposed Raman mode shifts are attributed to model limitations in capturing the underlying complexity of the electrochemical interface. Three approaches were followed to better estimate the vibrational frequencies: i) computation of additional adsorption geometries beyond the *bridge* configuration, ii) inclusion of $*NO_3$–$*H_2O$ co-adsorbates (**Figure 5d**), and iii) adsorption at undercoordinated sites of Ag_1 and Ag_3 for both $*NO_3$ (**Figure 5e**) and $*NO_2$ (**Figure S18a,b**). The nitrate adsorption configurations examined include $*NO_{3,fcc}$ (*i.e.*, monodentate adsorption at an *fcc* site) (**Figure S17a**) and $*NO_{3,flat}$ (**Figure S17b**). These structures provide useful reference for interpreting the experimentally observed vibrational frequencies and expected shifts for coexisting adsorption modes.

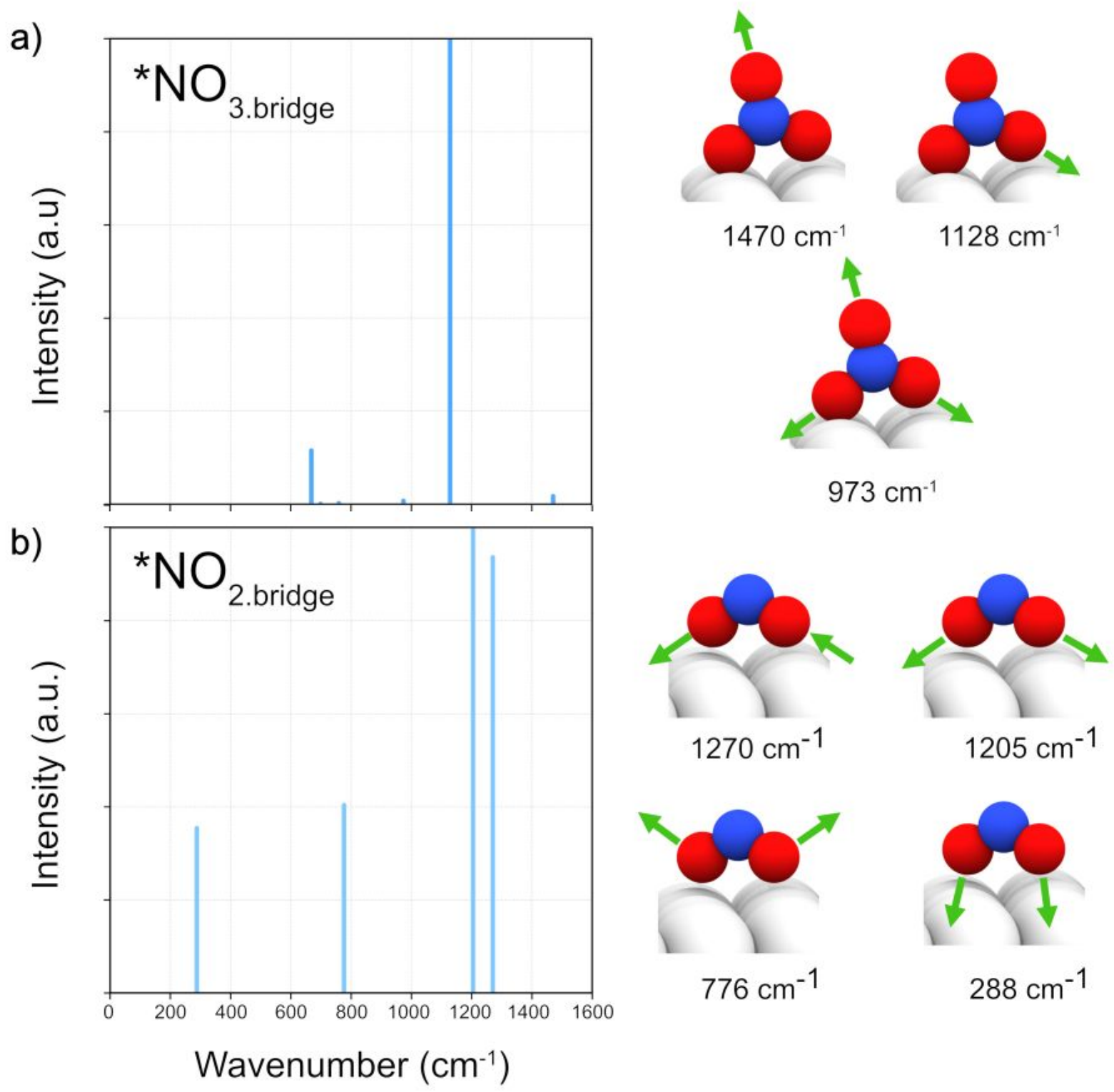

Figure S16. Computed nitrate and nitrite Raman spectra. Normalized, off-resonance activities of the DFT computed vibrational modes of (a) $*NO_{3,bridge}$ and (b) $*NO_{2,bridge}$. The green arrows represent each mode's displacement vectors.

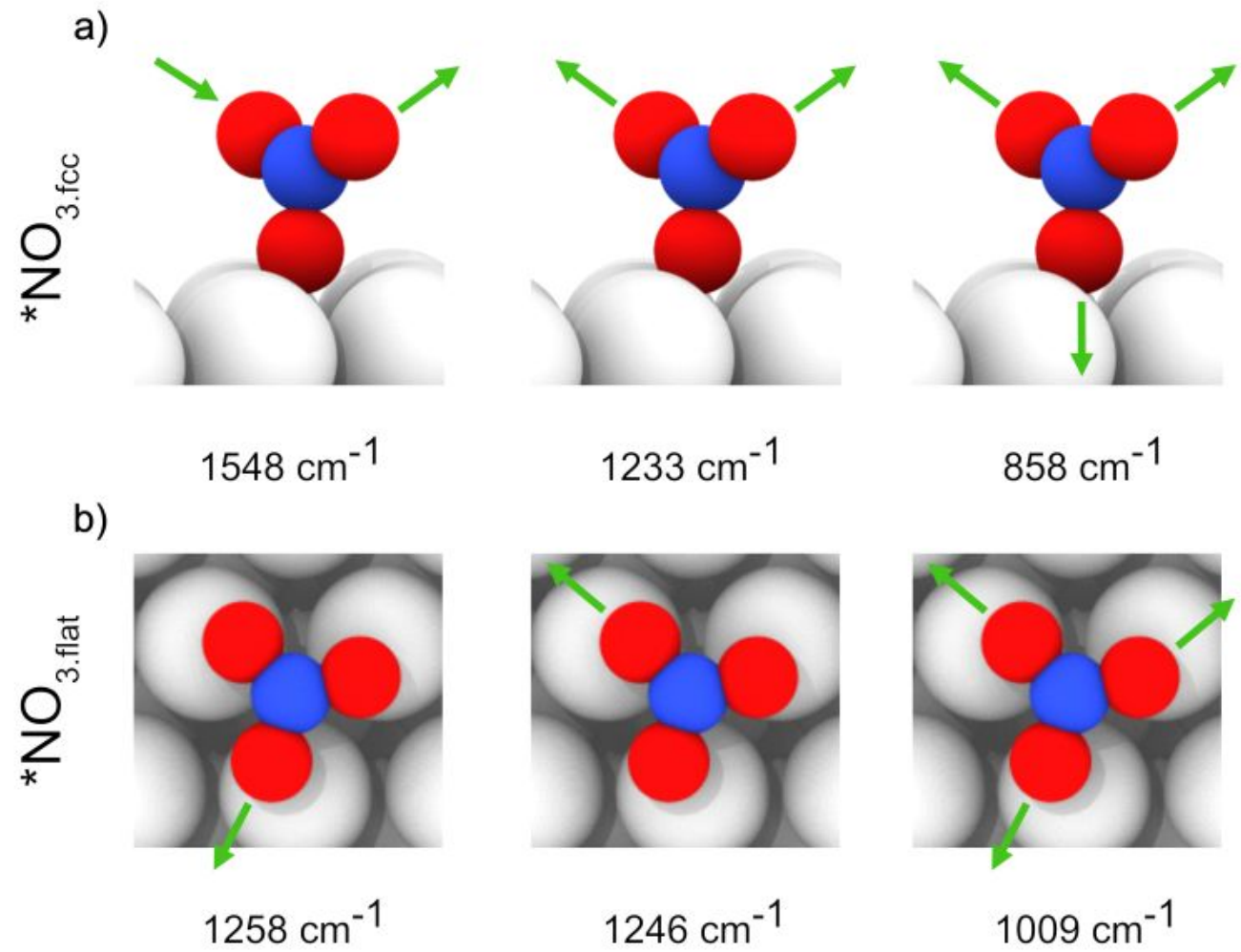

Figure S17. Additional nitrate vibrational modes. Highest-frequency stretching modes computed for nitrate adsorbed on Ag(111) in (a) $*NO_{3,fcc}$ and (b) $*NO_{3,flat}$ configurations. Green arrows indicate the displacement vectors of the vibrational modes.

Overall, the computed vibrational frequencies for $*NO_{2,bridge}$ show better agreement with experiment than those calculated for $*NO_{3,bridge}$. For the less stable O*NO* adsorption mode (**Figure 5c**), two distinct N–O stretching modes are predicted, spanning a frequency range of approximately 300 cm^{-1}. The highest-frequency mode is expected to be more sensitive to the interfacial environment because the corresponding N–O bond is oriented away from the surface and therefore interacts more strongly with the surrounding electrolyte. In contrast, the N–O bond oriented parallel to the surface exhibits a lower vibrational frequency due to stronger coupling with the surface atoms and a correspondingly larger reduced mass. The second N–O stretching mode appears at approximately 200 cm^{-1} higher frequency than the asymmetric stretching of $*NO_{2,bridge}$, indicating substantial decoupling from the surface.

Similar trends are observed for $*NO_2$ on the defective Ag surfaces. On the Ag_1 model (**Figure S18a**), adsorption partially decouples the two N–O stretching modes, increasing one frequency by 5–15 cm^{-1} while decreasing the other by less than 5 cm^{-1}. On the Ag_3 model (**Figure S18b**), the vibrational modes remain more strongly coupled to the surface, resulting in frequency shifts smaller than 5 cm^{-1} relative to the *bridge* configurations on the flat Ag(111) surface. Although the computed $*NO_{2,bridge}$ frequencies on the flat surface already agree reasonably well with experiment, adsorption on defective sites introduces additional variations in vibrational coupling that further illustrate the sensitivity of these modes to surface morphology.

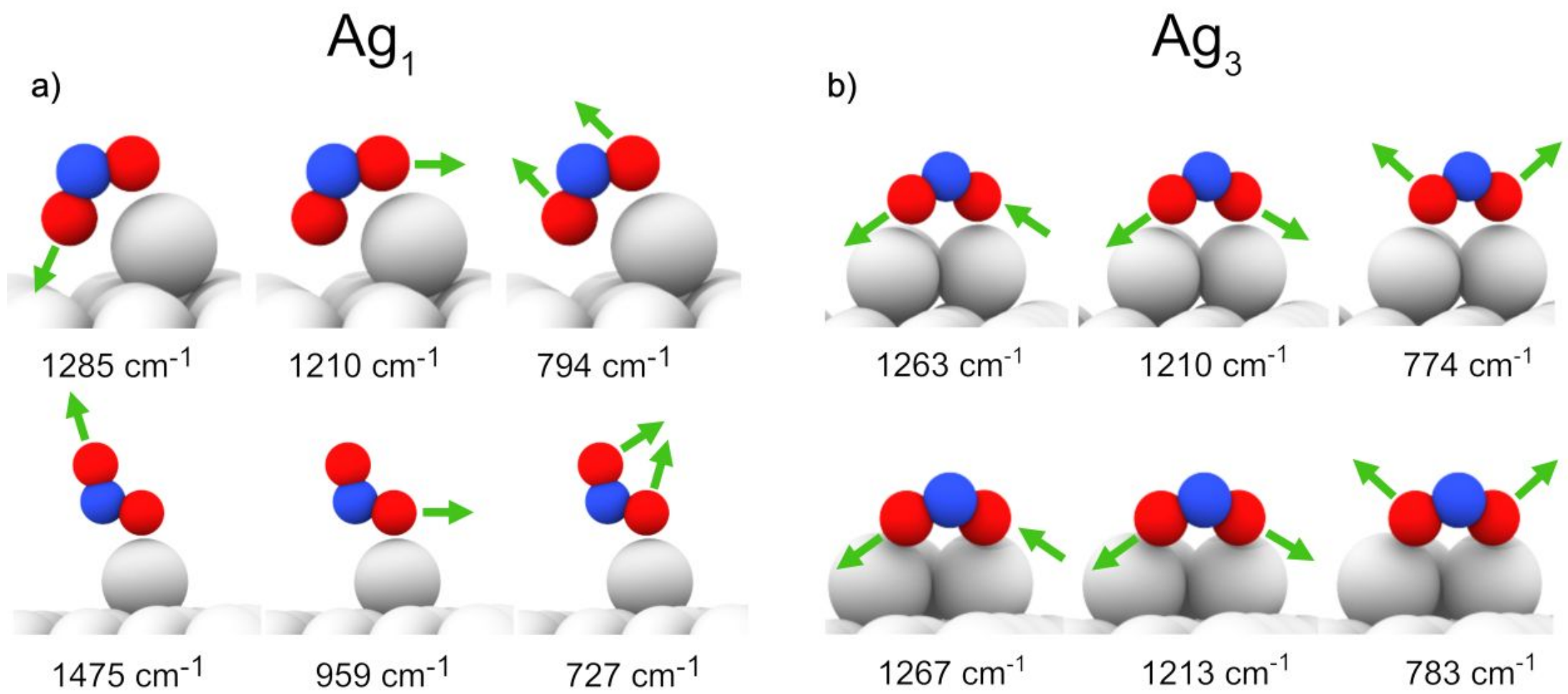

Figure S18. Nitrite vibrational modes on defective Ag surfaces. Highest-frequency stretching modes of $*NO_2$ computed for different adsorption configurations on (a) Ag_1 and (b) Ag_3 defective surface models. Green arrows represent the vibrational displacement vectors.

Regarding the Ag(211) stepped surface, the corresponding vibrational frequencies for the adsorbates shown in **Figures S15b** and **S15c** are summarized in **Table S1:**

Table S1. Calculated vibrational frequencies (cm^{-1}) for selected normal modes of $*NO_3$ and $*NO_2$ adsorbed on bare Ag(211) (**Figures S15b** and **S15c**, respectively). The corresponding frequencies calculated on Ag(111) are given in parentheses.

	$\overline{\nu_3}$	$\overline{\nu_2}$	$\overline{\nu_1}$
$*NO_3$	1486<br>(1470)	1132<br>(1128)	957<br>(973)
$*NO_2$	1270<br>(1270)	1209<br>(1205)	779<br>(776)

S3. References

(1) Ambrosio, R. C.; Gewirth, A. A. Characterization of Water Structure on Silver Electrode Surfaces by SERS with Two-Dimensional Correlation Spectroscopy. *Anal. Chem.* **2010**, *82* (4), 1305–1310. https://doi.org/10.1021/ac902299u

(2) Kirowa-Eisner, E.; Bonfil, Y.; Tzur, D.; Gileadi, E. Thermodynamics and Kinetics of Upd of Lead on Polycrystalline Silver and Gold. *Journal of Electroanalytical Chemistry* **2003**, *552*, 171–183. https://doi.org/10.1016/S0022-0728(03)00181-5

(3) Georgiev, D.; Pedersen, S. V.; Xie, R.; Fernández-Galiana, Á.; Stevens, M. M.; Barahona, M. RamanSPy: An Open-Source Python Package for Integrative Raman Spectroscopy Data Analysis. *Anal. Chem.* **2024**, *96* (21), 8492–8500. https://doi.org/10.1021/acs.analchem.4c00383

(4) Kresse, G.; Furthmüller, J. Efficient Iterative Schemes for *Ab Initio* Total-Energy Calculations Using a Plane-Wave Basis Set. *Phys. Rev. B* **1996**, *54* (16), 11169–11186. https://doi.org/10.1103/PhysRevB.54.11169

(5) Kresse, G.; Furthmüller, J. Efficiency of Ab-Initio Total Energy Calculations for Metals and Semiconductors Using a Plane-Wave Basis Set. *Comput. Mater. Sci.* **1996**, *6* (1), 15–50. https://doi.org/10.1016/0927-0256(96)00008-0

(6) Kohn, W.; Sham, L. J. Self-Consistent Equations Including Exchange and Correlation Effects. *Physical Review* **1965**, *140* (4A), A1133–A1138. https://doi.org/10.1103/PhysRev.140.A1133

(7) Hohenberg, P.; Kohn, W. Inhomogeneous Electron Gas. *Physical Review* **1964**, *136* (3B), B864–B871. https://doi.org/10.1103/PhysRev.136.B864

(8) Wellendorff, J.; Lundgaard, K. T.; Møgelhøj, A.; Petzold, V.; Landis, D. D.; Nørskov, J. K.; Bligaard, T.; Jacobsen, K. W. Density Functionals for Surface Science: Exchange-Correlation Model Development with Bayesian Error Estimation. *Phys. Rev. B* **2012**, *85* (23), 235149. https://doi.org/10.1103/PhysRevB.85.235149

(9) Klimeš, J.; Bowler, D. R.; Michaelides, A. Van Der Waals Density Functionals Applied to Solids. *Phys. Rev. B* **2011**, *83* (19), 195131. https://doi.org/10.1103/PhysRevB.83.195131

(10) Klimeš, J.; Bowler, D. R.; Michaelides, A. Chemical Accuracy for the van Der Waals Density Functional. *Journal of Physics: Condensed Matter* **2010**, *22* (2), 022201. https://doi.org/10.1088/0953-8984/22/2/022201

(11) Sharp, J.; Ciotti, A.; Andrews, H.; Udayasurian, S. R.; García-Melchor, M.; Li, T. Sustainable Electrosynthesis of Cyclohexanone Oxime through Nitrate Reduction on a Zn–Cu Alloy Catalyst. *ACS Catal.* **2024**, *14* (5), 3287–3297. https://doi.org/10.1021/acscatal.3c05388

(12) Ciotti, A.; García-Melchor, M. The Importance of Surface Coverages in the Rational Design of Electrocatalysts. *Curr. Opin. Electrochem.* **2023**, *42*, 101402. https://doi.org/10.1016/j.coelec.2023.101402

(13) Ciotti, A.; Rahaman, M.; Yeung, C. W. S.; Li, T.; Reisner, E.; García-Melchor, M. Driving Electrochemical Organic Hydrogenations on Metal Catalysts by Tailoring Hydrogen Surface Coverages. *J. Am. Chem. Soc.* **2025**, *147* (16), 13158–13168. https://doi.org/10.1021/jacs.4c15821

(14) Blöchl, P. E. Projector Augmented-Wave Method. *Phys. Rev. B* **1994**, *50* (24), 17953–17979. https://doi.org/10.1103/PhysRevB.50.17953

(15) Methfessel, M.; Paxton, A. T. High-Precision Sampling for Brillouin-Zone Integration in Metals. *Phys. Rev. B* **1989**, *40* (6), 3616–3621. https://doi.org/10.1103/PhysRevB.40.3616

(16) Hjorth Larsen, A.; Jørgen Mortensen, J.; Blomqvist, J.; Castelli, I. E.; Christensen, R.; Dułak, M.; Friis, J.; Groves, M. N.; Hammer, B.; Hargus, C.; Hermes, E. D.; Jennings, P. C.; Bjerre Jensen, P.; Kermode, J.; Kitchin, J. R.; Leonhard Kolsbjerg, E.; Kubal, J.; Kaasbjerg, K.; Lysgaard, S.; Bergmann Maronsson, J.; Maxson, T.; Olsen, T.; Pastewka, L.; Peterson, A.; Rostgaard, C.; Schiøtz, J.; Schütt, O.; Strange, M.; Thygesen, K. S.; Vegge, T.; Vilhelmsen, L.; Walter, M.; Zeng, Z.; Jacobsen, K. W. The Atomic Simulation Environment—a Python Library for Working with Atoms. *Journal of Physics: Condensed Matter* **2017**, *29* (27), 273002. https://doi.org/10.1088/1361-648X/aa680e

(17) Birch, F. Finite Elastic Strain of Cubic Crystals. *Physical Review* **1947**, *71* (11), 809–824. https://doi.org/10.1103/PhysRev.71.809

(18) Jain, A.; Ong, S. P.; Hautier, G.; Chen, W.; Richards, W. D.; Dacek, S.; Cholia, S.; Gunter, D.; Skinner, D.; Ceder, G.; Persson, K. A. Commentary: The Materials Project: A Materials Genome Approach to Accelerating Materials Innovation. *APL Mater.* **2013**, *1* (1). https://doi.org/10.1063/1.4812323

(19) Wang, V.; Xu, N.; Liu, J.-C.; Tang, G.; Geng, W.-T. VASPKIT: A User-Friendly Interface Facilitating High-Throughput Computing and Analysis Using VASP Code. *Comput. Phys. Commun.* **2021**, *267*, 108033. https://doi.org/10.1016/j.cpc.2021.108033

(20) Nørskov, J. K.; Rossmeisl, J.; Logadottir, A.; Lindqvist, L.; Kitchin, J. R.; Bligaard, T.; Jónsson, H. Origin of the Overpotential for Oxygen Reduction at a Fuel-Cell Cathode. *J. Phys. Chem. B* **2004**, *108* (46), 17886–17892. https://doi.org/10.1021/jp047349j

(21) Nørskov, J. K.; Bligaard, T.; Logadottir, A.; Kitchin, J. R.; Chen, J. G.; Pandelov, S.; Stimming, U. Trends in the Exchange Current for Hydrogen Evolution. *J. Electrochem. Soc.* **2005**, *152* (3), J23. https://doi.org/10.1149/1.1856988

(22) *https://github.com/raman-sc/VASP.*

(23) Brennan-Pollak, C.; Fenoll, D. A.; García-Melchor, M. When Do Constant-Charge and Constant-Potential Computational Simulations Agree? A Dipole-Based Framework for Predicting Electrochemical Surface Coverages. *Electrochim. Acta* **2026**, *566*, 148795. https://doi.org/10.1016/j.electacta.2026.148795
